# Stability theory of nano-fluid over an exponentially stretching cylindrical surface containing microorganisms

**DOI:** 10.1038/s41598-020-72545-y

**Published:** 2020-10-12

**Authors:** M. Ferdows, Md Amran Hossan Mojamder, M. Z. I. Bangalee, Shuyu Sun, Faris Alzahrani

**Affiliations:** 1 Department of Mathematics, King Abdulaziz University, Jeddah, 21589 Kingdom of Saudi Arabia; 2 Research Group of Fluid Flow Modeling and Simulation, Department of Applied Mathematics, University of Dhaka, Dhaka, 1000 Bangladesh; 3 Applied Mathematics and Computational Science Division, King Abdullah University of Science and Technology, Thuwal, 23955-6900 Kingdom of Saudi Arabia; 4 Department of Mathematics, College of Arts and Science, International University of Business and Agriculture and Technology, Uttara, Dhaka, 1230 Bangladesh

**Keywords:** Engineering, Mathematics and computing

## Abstract

This research is emphasized to describe the stability analysis in the form of dual solution of the flow and heat analysis on nanofluid over an exponential stretching cylindrical surface containing microorganisms. The research is also implemented to manifest the dual profiles of velocity, temperature and nanoparticle concentration in the effect of velocity ratio parameter ($s = \frac{{U_{w} }}{{U_{\infty } }}$). Living microorganisms’ cell are mixed into the nanofluid to neglect the unstable condition of nano type particles. The governing equations are transformed to non-linear ordinary differential equations with respect to pertinent boundary conditions by using similarity transformation. The significant differential equations are solved using build in function bvp4c in MATLAB. It is seen that the solution is not unique for vertical stretching sheet. This research is reached to excellent argument when found results are compared with available result. It is noticed that dual results are obtained demanding on critical value ($s_{c}$), the meanings are indicated at these critical values both solutions are connected and behind these critical value boundary layer separates thus the solution are not stable.

## Introduction

The flow profile conveyed by viscous fluid between to coaxially rotated disk is surveyed in Majeed et al.^1^. Heat and mass transfer profile of second grad fluid over an inclined cylinder with diffusion heat flux described by Bilal et al.^2^. The features of Carreau flow and important aspect of thermal stratification are concluded by Bilal et al.^3^. The momentum and heat transfer of electro-magneto hydrodynamics boundary layer flow are incorporated in Bilal et al.^4^ over stretching sheet with slip. Analysis of Newtonian flow and the flow of power law fluid are manifested by Mahmood et al.^5^ with the feature of shear thinning and shear thickening. The flow features of power law materials with channel driven cavity configuration investigated by Mahmood et al.^6^. The pseudo plastic and dilatant materials have extensive applications on metallurgical processes. Characteristics of power law fluid were addressed in Mahmood et al.^7^ with the attributes of pseudo plastic and dilatant materials in channel driven cavity.

The aspects of temperature dependent dynamic viscosity of Maxwell fluid are obtained over a variable thicken surface by Khan et al.^8^. Bio convection MHD Carreau Nano fluid flow and thermo physical aspects of MHD were focused respectively on Khan et al.^9^ and Hussain et al.^10^ and model have been constructed by Fourier’s and Fick’s laws. The features of MHD Prandtl-Eyring Nano fluid over stretching surface introduced by Rehman et al.^11^ with the effect of Navier slip and convective boundary condition. The Maxwell fluid flow of heat and mass transfer over stretching sheet were explicitly drawn with solar radiation and viscous desperation by Khan et al.^12^. The heat and mass diffusion of Maxwell Nano fluid over stretching surface near stagnation point incorporated in Khan et al.^13^ and are implied by Fourier’s and Fick’s laws.

The Bio-convection boundary layer flow and nanofluid model were introduced in Buongiorno^14^. Several similar works of nanofluid and heat rate were done on^14–18^. Advance analysis of applications of nanofluid have been reviewed in Refs.^19–22^ and many others have been analyzed to enhance nanofluid effect over heat transfer with the use of parameters. Buongiorno’s model^14^ and the Tiwari-Das model^23^ are two familiar method for the analysis of nanofluid which have been worked by researchers. In Buongiorno model, the total fluid velocity and the relative/slip velocity were counted as the nanofluid velocity. This model also scrutinized the effect of parameter as Brownian diffusion and thermophoresis. In opposition of Buongiorno’s model, the solid volume fractions of the nanoparticles were introduced by Tiwari-Das model et al.^23^. By the characteristic of nanofluid flows Brownian diffusion and thermophoresis are the most important parameters implies by Ref.^14^. Present study of Refs.^24–29^ add nanofluid in the convective boundary layer flow. Nano-polymer stretching flows with radioactive magneto hydrodynamics were investigated by Ferdows et al.^30^. Numerical studies of magnetic Nano-bio-polymerswere done by Uddin et al.^31^.

To create the bio convection process add microorganism with the base fluid. Bioconvection could cause an unstable density profile of the fluid, if the density of microorganism is seen to be greater than the free stream fluid which is followed by Raees et al.^32^. Microorganisms survive to base fluid if the base fluid is water and remain stable in the nanofluid suspension for a few of weeks by Anoop et al.^33^. Nanoparticles could multiply the nanofluid’s viscosity and tends to accelerate bio convection instability^34^. Nano-fluidon boundary layer flow, stretching cylinder, containing microorganism and bio-convection have been described respectively by Refs.^35–38^. The existence of dual solutions for conducting flow and mixed convection boundary layer flow with suction and injection are analyzed by Ishak et al.^39^. In study on Newtonian fluids boundary layer is contrasting to the free stream flow then multiple solutions would be found on Ishak et al.^40^. Najib et al.^41^ is analyzed the dual solutions exists over stretching cylinder along with mass suction. The researchers such as Refs.^42–44^ have scrutinized the stability theory into their research to ensure the flow is stable and have meaningful solution. The article is mainly cover on expending research work by Refs.^42–48^ to verify the existence of the dual (first solution and second solution) solutions with the consideration of different parameter. The stable solution will be noticed when we get the dual or multiple solutions and with the help of numerical analysis to verify which solution is stable or not.

### Problem formulation

Consider circular cylinder of radius a which is stretching exponentially along with velocity $U_{w}$. $T_{w}$, $C_{w}$, and $m_{w}$ are the constant temperature, constant nanoparticle concentration and constant density of microorganism respectively at the surface of the cylinder. The boundary layer flow contains with nanoparticles and microorganism is flowing over this cylinder. The uniform ambient temperature, ambient nanoparticle concentration and ambient density of microorganism are $T_{\infty }$, $C_{\infty }$, $m_{\infty }$ respectively. $T_{w} - T_{\infty } > 0$ is the quantity for conducting flow, while $T_{w} - T_{\infty } < 0$ is the quantity contrasting flow.

**Figure 1:**
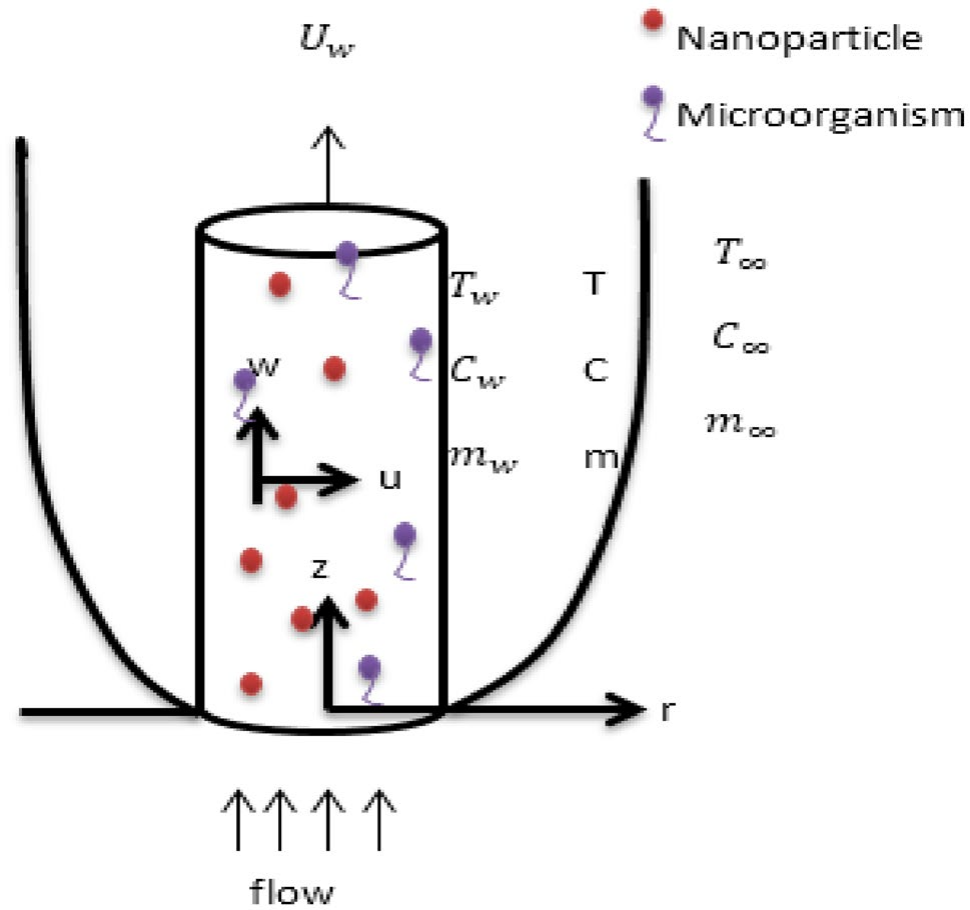
Physical Significant Model and fluid coordination.

Consider velocity component along the $\left( {r,z} \right)$ axes are $\left( {u,w} \right)$. The z coordinate system considered along the stretching cylinder and $r$ coordinate normal to the cylindrical surface (see Fig. 1). The uniform velocity $U_{w}$ is moving along z direction and $u = 0$ so there is no velocity along r direction so that u momentum equation omitted.

The continuity equation for the nanoparticles in the absence of chemical reactions is

$$\frac{\partial \phi }{{\partial t}} + v \cdot \nabla \phi = - \frac{1}{{\rho_{p} }}\nabla \cdot j_{p}$$

where $t$ is time, $j_{p}$ is the diffusion mass flux. If external forces negligible $j_{p}$ can be sum of two diffusion terms i.e. Brownian diffusion and thermophoresis diffusion

$$j_{p} = j_{p,B} + j_{p,T} = - \rho_{p} D_{B} \nabla \phi - \rho_{p} D_{T} \frac{\nabla T}{T}$$

$$\frac{\partial \phi }{{\partial t}} + v \cdot \nabla \phi = \nabla \cdot \left[ {D_{B} \nabla \phi + D_{T} \frac{\nabla T}{T}} \right]$$

Equation states that the nanoparticles can move homogeneously with the fluid (second term of the left-hand side), but they also possess a slip velocity relatively to the fluid (right-hand side), which is due to Brownian diffusion and thermophoresis.

The assumptions from the model, the governing equations are the conservation of total mass, momentum, thermal energy, nanoparticle concentration and density of microorganism which can be written as^36^:

1 $$\frac{\partial u}{{\partial r}} + \frac{u}{r} + \frac{\partial w}{{\partial z}} = 0$$

2 $$u\frac{\partial w}{{\partial r}} + w\frac{\partial w}{{\partial z}} = - \frac{1}{\rho }\frac{\partial p}{{\partial z}} + \vartheta \left( {\frac{{\partial^{2} w}}{{\partial r^{2} }} + \frac{1}{r}\frac{\partial w}{{\partial r}}} \right) + \left[ {\frac{1}{\rho }\left( {\rho^{*} - \rho } \right)\left( {\varphi - \varphi_{\infty } } \right) + \left( {1 - \varphi_{\infty } } \right)\beta \left( {T - T_{\infty } } \right)} \right]g - \frac{{\vartheta \varphi_{p} }}{{k_{0} }}w$$

3 $$u\frac{\partial T}{{\partial r}} + w\frac{\partial T}{{\partial Z}} = \alpha \left( {\frac{{\partial^{2} T}}{{\partial r^{2} }} + \frac{1}{r}\frac{\partial T}{{\partial r}}} \right) + \tau \left[ {D_{B} \frac{\partial T}{{\partial r}}\frac{\partial \varphi }{{\partial r}} + \frac{{D_{T} }}{{T_{\infty } }}\left( {\frac{\partial T}{{\partial r}}} \right)^{2} } \right]$$

4 $$u\frac{\partial C}{{\partial r}} + w\frac{\partial C}{{\partial Z}} = D_{B} \left( {\frac{{\partial^{2} C}}{{\partial r^{2} }} + \frac{1}{r}\frac{\partial C}{{\partial r}}} \right) + \frac{{D_{T} }}{{T_{\infty } }}\left( {\frac{{\partial^{2} T}}{{\partial r^{2} }} + \frac{1}{r}\frac{\partial T}{{\partial r}}} \right)$$

5 $$u\frac{\partial m}{{\partial r}} + w\frac{\partial m}{{\partial Z}} = D_{n} \left( {\frac{{\partial^{2} m}}{{\partial r^{2} }} + \frac{1}{r}\frac{\partial m}{{\partial r}}} \right) - \frac{{bW_{\varphi } }}{{C_{w} - C_{\infty } }}\left[ {\frac{\partial }{\partial r}\left( {n\frac{\partial C}{{\partial r}}} \right)} \right]$$

From above u and $w$ are the velocity with respect to r and z axes, where $\vartheta$ is the viscosity, p is the pressure, g is the gravitational acceleration along z direction,$\rho$ is the density,$\beta$ is the coefficient of thermal expansion, $k_{0}$ is the permeability of porous space, $C_{p}$ is the porosity of porous space, $T$ is the temperature,$\alpha$ is the thermal diffusivity , $\tau = \frac{{\left( {\rho c} \right)_{p} }}{{\left( {\rho c} \right)_{f} }}$ is a parameter with $\left( {\rho c} \right)_{p}$ heat capacity of nanoparticle, $\left( {\rho c} \right)_{f}$ being heat capacity of fluid, $D_{B}$ is the Brownian diffusion coefficient, $D_{T}$ is the thermophoretic diffusion coefficient, $C$ is the nanoparticle concentration function,$D_{n}$ being the diffusivity of microorganisms, b being the constant and $W_{c}$ is cell moving speed.

Boundary conditions for the above problem are:

6 $$u = 0,w = U_{w} = 2ake^{\frac{z}{a}} ,T = T_{w} , C = C_{w} , m = m_{w} , at r = a$$

7 $$w \to 0, T \to T_{\infty } ,C \to C_{\infty } , m \to m_{\infty } , at r \to \infty$$

Introduce the following similarity transformations (see Rehman et al.^35^)

8 $$u = - ake^{\frac{z}{a}} \frac{f\left( \eta \right)}{{\sqrt \eta }}, w = 2ake^{\frac{z}{a}} f^{\prime}\left( \eta \right)$$

9 $$\theta \left( \eta \right) = \frac{{T - T_{\infty } }}{{T_{w} - T_{\infty } }}, \phi \left( \eta \right) = \frac{{C - C_{\infty } }}{{C_{w} - C_{\infty } }}, \chi \left( \eta \right) = \frac{{m - m_{\infty } }}{{m_{w} - m_{\infty } }}, \eta = \frac{{r^{2} }}{{a^{2} }}$$

where the temperature, nanoparticle concentration differences and density of microorganism differences are calculated from the relations $T_{w} - T_{\infty } = ce^{\frac{z}{a}}$, $C_{w} - C_{\infty } = ce^{\frac{z}{a}}$, $m_{w} - m_{\infty } = ce^{\frac{z}{a}}$.

Substituting (8) and (9) into Eqs. (2)–(5) we obtained

10 $$\frac{1}{Re}\left( {\eta f^{\prime\prime\prime} + f^{\prime\prime}} \right) + ff^{\prime\prime} - f^{{\prime}2} + \lambda \left( {1 - C_{\infty } } \right)\left( {\theta + N_{r} \phi } \right) - k_{p} f^{\prime} = 0$$

11 $$\frac{1}{Re \cdot Pr}\left( {\eta \theta^{\prime\prime} + \theta^{\prime}} \right) + f\theta^{\prime} - f^{\prime}\theta + \frac{{N_{b} }}{Re \cdot Pr} \cdot \eta \theta^{\prime}\phi^{\prime} + \frac{{N_{T} }}{Re \cdot Pr} \cdot \eta \theta^{{\prime}2} = 0$$

12 $$\frac{1}{Re \cdot Sc}\left( {\eta \cdot \phi^{\prime\prime} + \phi^{\prime}} \right) + f\phi^{\prime} - f^{\prime}\phi + \frac{1}{Re \cdot Sc} \cdot \frac{{N_{T} }}{{N_{b} }}\left( {\eta \theta^{\prime\prime} + \theta^{\prime}} \right) = 0$$

13 $$\frac{1}{Re \cdot Sb}\left( {\eta \cdot \chi^{\prime\prime} + \chi^{\prime}} \right) + f\chi^{\prime} - f^{\prime}\chi + \frac{Pe}{{Re \cdot Sb}} \cdot \left( {\eta \chi \phi^{\prime\prime} + \frac{\chi }{2}\phi^{\prime} + \eta \chi^{\prime}\phi^{\prime}} \right) = 0$$

The transformed boundary condition become

14 $$f\left( 1 \right) = 0 \quad f^{\prime}\left( 1 \right) = s\quad f^{\prime}\left( \infty \right) = 0$$

15 $$\theta \left( 1 \right) = 1 \quad \theta \left( \infty \right) = 0$$

16 $$\phi \left( 1 \right) = 1 \quad \phi \left( \infty \right) = 0$$

17 $$\chi \left( 1 \right) = 1 \quad \chi \left( \infty \right) \to 0$$

where

$$Re = \frac{{aU_{w} }}{4\vartheta } =$$ Reynolds number.

$\lambda = \frac{{g\beta a\left( {T_{w} - T_{\infty } } \right)}}{{U_{w}^{2} }} =$ Natural convection parameter.

$$k_{p} = \frac{{\vartheta aC_{p} }}{{U_{w} k_{0} }} =$$ Porosity parameter.

$$N_{r} = \frac{{\left( {\rho^{*} - \rho } \right)\left( {C_{w} - C_{\infty } } \right)}}{{\beta \rho \left( {1 - C_{\infty } } \right)\left( {T_{w} - T_{\infty } } \right)}} =$$ Buoyancy ratio.

$$Pr = \frac{\vartheta }{\alpha } =$$ Prandtl number.

$N_{b} = \frac{{\tau D_{B} \left( {C_{w} - C_{\infty } } \right)}}{\alpha } =$ Brownian motion parameter.

$$N_{T} = \frac{{\tau D_{T} \left( {T_{w} - T_{\infty } } \right)}}{{\alpha T_{\infty } }} =$$ Thermophoresis parameter,

$$S_{c} = \frac{\vartheta }{{D_{B} }} =$$ Schmidt number.

$$P_{e} = \frac{{bW_{C} }}{{D_{n} }} =$$ Peclet number.

$S_{b} = \frac{\vartheta }{{D_{n} }} =$ Bioconvection Schmidt number.

### Stability analysis

This works, we showed that for the certain range of parameter s the multiple solutions are possible and we analyzed whether the solution is stable or not. For this reason we take new dimensionless variable $\delta$, where $\delta$ cause to begin an initial value problem and consistent. The unsteady problem arises for stability analysis from our considered steady formula:

18 $$\frac{\partial w}{{\partial t}} + u\frac{\partial w}{{\partial r}} + w\frac{\partial w}{{\partial z}} = - \frac{1}{\rho }\frac{\partial p}{{\partial z}} + \vartheta \left( {\frac{{\partial^{2} w}}{{\partial r^{2} }} + \frac{1}{r}\frac{\partial w}{{\partial r}}} \right) + \left[ {\frac{1}{\rho }\left( {\rho^{*} - \rho } \right)\left( {\varphi - \varphi_{\infty } } \right) + \left( {1 - \varphi_{\infty } } \right)\beta \left( {T - T_{\infty } } \right)} \right]g - \frac{{\vartheta \varphi_{p} }}{{k_{0} }}w$$

19 $$\frac{\partial T}{{\partial t}} + u\frac{\partial T}{{\partial r}} + w\frac{\partial T}{{\partial Z}} = \alpha \left( {\frac{{\partial^{2} T}}{{\partial r^{2} }} + \frac{1}{r}\frac{\partial T}{{\partial r}}} \right) + \tau \left[ {D_{B} \frac{\partial T}{{\partial r}}\frac{\partial \varphi }{{\partial r}} + \frac{{D_{T} }}{{T_{\infty } }}\left( {\frac{\partial T}{{\partial r}}} \right)^{2} } \right]$$

20 $$\frac{\partial C}{{\partial t}} + u\frac{\partial C}{{\partial r}} + w\frac{\partial C}{{\partial Z}} = D_{B} \left( {\frac{{\partial^{2} C}}{{\partial r^{2} }} + \frac{1}{r}\frac{\partial C}{{\partial r}}} \right) + \frac{{D_{T} }}{{T_{\infty } }}\left( {\frac{{\partial^{2} T}}{{\partial r^{2} }} + \frac{1}{r}\frac{\partial T}{{\partial r}}} \right)$$

21 $$\frac{\partial m}{{\partial t}} + u\frac{\partial m}{{\partial r}} + w\frac{\partial m}{{\partial Z}} = D_{n} \left( {\frac{{\partial^{2} m}}{{\partial r^{2} }} + \frac{1}{r}\frac{\partial m}{{\partial r}}} \right) - \frac{{bW_{\varphi } }}{{C_{w} - C_{\infty } }}\left[ {\frac{\partial }{\partial r}\left( {n\frac{\partial C}{{\partial r}}} \right)} \right]$$

Introducing similarity variables as

22 $$u = - ake^{\frac{z}{a}} \frac{{f\left( {\eta ,\delta } \right)}}{\sqrt \eta }, w = 2ake^{\frac{z}{a}} f^{\prime}\left( {\eta ,\delta } \right)$$

23 $$\theta \left( {\eta ,\delta } \right) = \frac{{T - T_{\infty } }}{{T_{w} - T_{\infty } }}, \phi \left( {\eta ,\delta } \right) = \frac{{C - C_{\infty } }}{{C_{w} - C_{\infty } }}, \chi \left( {\eta ,\delta } \right) = \frac{{m - m_{\infty } }}{{m_{w} - m_{\infty } }}, \eta = \frac{{r^{2} }}{{a^{2} }},\delta = \frac{{U_{w} t}}{a}$$

So the converted equation can be written as

24 $$\frac{1}{Re}\left( {\eta f_{\eta \eta \eta } + f_{\eta \eta } } \right) + ff_{\eta \eta } - f_{\eta }^{2} + \lambda \left( {1 - C_{\infty } } \right)\left( {\theta + N_{r} \phi } \right) - k_{p} f_{\eta } - f_{\eta \delta } = 0$$

25 $$\frac{1}{Re \cdot Pr}\left( {\eta \theta_{\eta \eta } + \theta_{\eta } } \right) + f\theta_{\eta } - f_{\eta } \theta + \frac{{N_{b} }}{Re \cdot Pr} \cdot \eta \theta_{\eta } \phi_{\eta } +\frac{{N_{T} }}{Re \cdot Pr} \cdot \eta \theta_{\eta }^{2} - \theta_{\delta } = 0$$

26 $$\frac{1}{Re \cdot Sc}\left( {\eta \phi_{\eta \eta } + \phi_{\eta } } \right) + f\phi_{\eta } - f_{\eta } \phi + \frac{1}{Re \cdot Sc} \cdot \frac{{N_{T} }}{{N_{b} }}\left( {\eta \theta_{\eta \eta } + \theta_{\eta } } \right) - \phi_{\delta } = 0$$

27 $$\frac{1}{Re \cdot Sb}\left( {\eta \cdot \chi_{\eta \eta } + \chi_{ \eta } } \right) + f\chi_{ \eta } - f_{\eta } \chi + \frac{Pe}{{Re \cdot Sb}} \cdot \left( {\eta \chi \phi_{\eta \eta } + \frac{\chi }{2}\phi_{\eta } + \eta \chi_{ \eta } \phi_{\eta } } \right) - \chi_{\delta } = 0$$

And are subjected to the boundary conditions

28 $$f\left( {1,\delta } \right) = 0, f_{\eta } \left( {1,\delta } \right) = s, \theta \left( {1,\delta } \right) = 1, \phi \left( {1,\delta } \right) = 1, \chi \left( {1,\delta } \right) = 1$$

29 $$f_{\eta } \left( {\eta ,\delta } \right) \to 0, \theta \left( {\eta ,\delta } \right) \to 0, \phi \left( {\eta ,\delta } \right) \to 0, \chi \left( {\eta ,\delta } \right) \to 0 \quad as \quad \eta \to \infty$$

To check the stability of the steadiness solution, we take $f\left( \eta \right) = f_{0} \left( \eta \right)$, $\theta \left( \eta \right) = \theta_{0} \left( \eta \right)$, $\phi \left( \eta \right) = \phi_{0} \left( \eta \right)$, and $\chi \left( \eta \right) = \chi_{0} \left( \eta \right)$ which fulfilling the boundary value problem (1)–(7)

$$f\left( {\eta ,\delta } \right) = f_{0} \left( \eta \right) + e^{ - l\delta } F\left( {\eta ,\delta } \right)$$

$$\theta \left( {\eta ,\delta } \right) = \theta_{0} \left( \eta \right) + e^{ - l\delta } G\left( {\eta ,\delta } \right)$$

$$\phi \left( {\eta ,\delta } \right) = \phi_{0} \left( \eta \right) + e^{ - l\delta } H\left( {\eta ,\delta } \right)$$

$$\chi \left( {\eta ,\delta } \right) = \chi_{0} \left( \eta \right) + e^{ - l\delta } I\left( {\eta ,\delta } \right)$$

where $l$ is an eigenvalue, and $F\left( {\eta ,\delta } \right)$, $G\left( {\eta ,\delta } \right)$, $H\left( {\eta ,\delta } \right)$, $I\left( {\eta ,\delta } \right)$ are small relative to $f_{0} \left( \eta \right)$, $\theta_{0} \left( \eta \right)$, $\phi_{0} \left( \eta \right)$, $\chi_{0} \left( \eta \right)$. Substituting these in (24)–(29) we have

30 $$\frac{1}{Re}\left( {\eta F^{\prime\prime\prime} + F^{\prime\prime}} \right) + fF^{\prime\prime} + Ff^{\prime\prime} - 2f^{\prime}F^{\prime} + \lambda \left( {1 - C_{\infty } } \right)\left( {G + N_{r} H} \right) - k_{p} F^{\prime} + lF^{\prime} = 0$$

31 $$\frac{1}{Re \cdot Pr}\left( {\eta G^{\prime\prime} + G^{\prime}} \right) + fG^{\prime} + F\theta^{\prime} - f^{\prime}G - F^{\prime}\theta + \frac{{N_{b} }}{Re \cdot Pr}\eta (\theta^{{\prime}} H^{\prime} + G^{\prime}\phi ^{\prime}) + 2\frac{{N_{T} }}{Re \cdot Pr}\eta \theta ^{\prime}G^{\prime} + lG = 0$$

32 $$\frac{1}{Re \cdot Sc}\left( {\eta \cdot H^{\prime\prime} + H^{\prime}} \right) + fH^{\prime} + F\phi^{\prime} - f^{\prime}H - F^{\prime}\phi + \frac{1}{Re \cdot Sc} \cdot \frac{{N_{T} }}{{N_{b} }}\left( {\eta G^{\prime\prime} + G^{\prime}} \right) + lH = 0$$

33 $$\frac{1}{Re \cdot Sb}\left( {\eta I^{\prime\prime} + I^{\prime}} \right) + fI^{\prime} + F\chi^{\prime} - f^{\prime}I - F^{\prime}\chi + \frac{Pe}{{Re \cdot Sb}} \cdot [\eta \left( {\chi H^{\prime\prime} + I\phi^{\prime\prime}} \right) + \frac{1}{2}(\chi H^{{\prime}} + I\phi^{\prime}) + \eta (I^{{\prime}} \phi^{\prime} + \chi ^{\prime}H^{\prime})] = 0$$

With respect to the boundary conditions

34 $$F\left( {0,\delta } \right) = 0, F_{\eta } \left( {0,\delta } \right) = 0, G\left( {0,\delta } \right) = 0, H\left( {0,\delta } \right) = 0, I\left( {0,\delta } \right) = 0$$

35 $$F_{\eta } \left( {\infty ,\delta } \right) \to 0, G\left( {\infty ,\delta } \right) \to 0, H\left( {\infty ,\delta } \right) \to 0, I\left( {\infty ,\delta } \right) \to 0$$

By setting $\delta = 0$, the solutions $f\left( \eta \right) = f_{0} \left( \eta \right)$, $\theta \left( \eta \right) = \theta_{0} \left( \eta \right)$, $\phi \left( \eta \right) = \phi_{0} \left( \eta \right)$, $\chi \left( \eta \right) = \chi_{0} \left( \eta \right)$ of the steady Eqs. (1)–(7) are obtained.

## Numerical method

In the context of bvp4c function described in MATLAB, we need to transform the higher order nonlinear ordinary differential equations to first order ordinary differential equations. From this technique, with the diversity of initial guess of $f^{\prime}$,$f^{\prime\prime}$, $\theta$, $\theta ^{\prime}$, $\phi$, $\phi ^{\prime}$, $\chi$, $\chi ^{\prime}$ we can able to find the first and second solution. So the Eqs. (10)–(13) become

$$f^{\prime\prime\prime} = \frac{1}{\eta }\left[ {Re\left( {f^{{\prime}2} - ff^{\prime\prime} - \lambda \left( {1 - C_{\infty } } \right)\left( {\theta + N_{r} \phi } \right) + k_{p} f^{\prime}} \right) - f^{\prime\prime}} \right]$$

$$\theta^{\prime\prime} = \frac{1}{\eta }\left[ {R_{e} P_{r} \left( {f^{\prime}\theta - f\theta^{\prime}} \right) - \eta N_{b} \theta^{\prime}\phi^{\prime} - \eta N_{T} \theta^{{\prime}2} - \theta ^{\prime}} \right]$$

$$\phi^{\prime\prime} = \frac{1}{\eta }\left[ {R_{e} S_{c} \left( {f^{\prime}\phi - f\phi^{\prime}} \right) - \frac{{N_{T} }}{{N_{b} }}\left( {R_{e} P_{r} \left( {f^{\prime}\theta - f\theta^{\prime}} \right) - \eta N_{b} \theta^{\prime}\phi^{\prime} - \eta N_{T} \theta^{{\prime}2} } \right) - \phi ^{\prime}} \right]$$

$$\chi^{\prime\prime} = \frac{1}{\eta }\left[ {R_{e} S_{b} \left( {f^{\prime}\chi - f\chi^{\prime}} \right) + P_{e} \left( {\chi \left( {R_{e} S_{c} \left( {f^{\prime}\phi - f\phi^{\prime}} \right) - \frac{{N_{T} }}{{N_{b} }}\left( {R_{e} P_{r} \left( {f^{\prime}\theta - f\theta^{\prime}} \right) - \eta N_{b} \theta^{\prime}\phi^{\prime} - \eta N_{T} \theta^{{\prime}2} } \right) - \phi^{\prime}} \right) + \frac{1}{2}\chi \phi^{\prime} + \eta \chi^{\prime}\phi^{\prime}} \right) - \chi ^{\prime}} \right]$$

Now we need to transform this above equation into first order differential equation. For this let $\eta = x$ and

$$y_{1} = f, y_{2} = f^{\prime}, y_{3} = f^{\prime\prime}$$

$$y_{4} = \theta , y_{5} = \theta^{\prime}, y_{6} = \phi ,$$

$$y_{7} = \phi^{\prime}, y_{8} = \chi , y_{9} = \chi ^{\prime}$$

The corresponding first order differential equations are

$$\frac{{dy_{1} }}{dx} = f^{\prime} = y_{2}$$

$$\frac{{dy_{2} }}{dx} = f^{\prime\prime} = y_{3}$$

$$\frac{{dy_{3} }}{dx} = f^{\prime\prime\prime} = \frac{1}{x}\left[ {Re\left( {y_{2}^{2} - y_{1} y_{3} - \lambda \left( {1 - C_{\infty } } \right)\left( {y_{4} + N_{r} y_{6} } \right) + k_{p} y_{2} } \right) - y_{3} } \right]$$

$$\frac{{dy_{4} }}{dx} = \theta^{\prime} = y_{5}$$

$$\frac{{dy_{5} }}{dx} = \theta^{\prime\prime} = \frac{1}{x}\left[ {R_{e} P_{r} \left( {y_{2} y_{4} - y_{1} y_{5} } \right) - \eta N_{b} y_{5} y_{7} - \eta N_{T} y_{5}^{2} - y_{5} } \right]$$

$$\frac{{dy_{6} }}{dx} = \phi^{\prime} = y_{7}$$

$$\frac{{dy_{7} }}{dx} = \phi^{\prime\prime} = \frac{1}{x}\left[ {R_{e} S_{c} \left( {y_{2} y_{6} - y_{1} y_{7} } \right) - \frac{{N_{T} }}{{N_{b} }}\left( {R_{e} P_{r} \left( {y_{2} y_{4} - y_{1} y_{5} } \right) - \eta N_{b} y_{5} y_{7} - \eta N_{T} y_{5}^{2} } \right) - y_{7} } \right]$$

$$\frac{{dy_{8} }}{dx} = \chi^{\prime} = y_{9}$$

$$\frac{{dy_{9} }}{dx} = \chi^{\prime\prime} = \frac{1}{x}\left[ {R_{e} S_{b} \left( {y_{2} y_{8} - y_{1} y_{9} } \right) + P_{e} \left( {y_{8} \left( {R_{e} S_{c} \left( {y_{2} y_{6} - y_{1} y_{7} } \right) - \frac{{N_{T} }}{{N_{b} }}\left( {R_{e} P_{r} \left( {y_{2} y_{4} - y_{1} y_{5} } \right) - \eta N_{b} y_{5} y_{7} - \eta N_{T} y_{5}^{2} } \right) - y_{7} } \right) + \frac{1}{2}y_{8} y_{7} + \eta y_{9} y_{7} } \right) - y_{9} } \right]$$

We need to transform the boundary conditions (14)-(17) and let $ya$ be the left boundary, $yb$ be the right boundary then

$$ya\left( 1 \right) = 0, ya\left( 2 \right) - s = 0, yb\left( 2 \right) = 0$$

$$ya\left( 4 \right) - 1 = 0, yb\left( 4 \right) = 0$$

$$ya\left( 6 \right) - 1 = 0, yb\left( 6 \right) = 0$$

$$ya\left( 8 \right) - 1 = 0, yb\left( 8 \right) = 0$$

## Results

**Figure 2:**
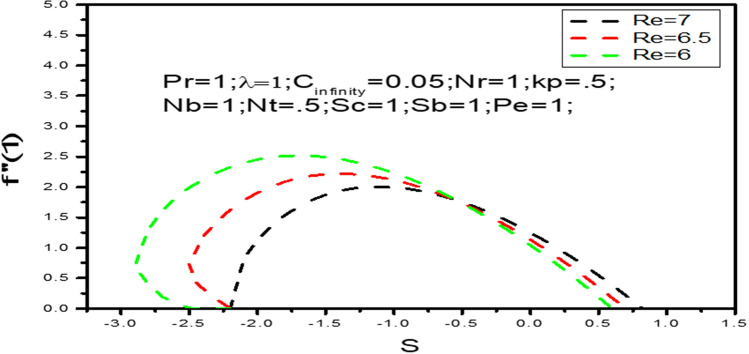
Local Skin friction coefficient $f^{\prime\prime}\left( 1 \right)$ with $s$ when $Re = 6,6.5,7$

We variety the skin friction coefficient $f^{\prime\prime}\left( 1 \right)$ along $s$ in Fig. 2 for several values of $R_{e}$. This Figure shows that multiple solutions are possible For values of $R_{e}$ when $s > 0$, the multiple solution exist. For example dual solution are obtained for $R_{e} = 6$, when $s > - 2.9 = s_{c}$, and for $s < s_{c}$ there are no solution or unique solution exist for $R_{e} = 6$. Similarly dual solution can be simulated for $Re = 6.5$ when $s > - 2.5 = s_{c}$ and $s < s_{c}$ there is no solution or unique solution may exist. At these critical values of $s \left( {say\ s_{c} } \right)$ thus the unique solution is possible and connect the both branches. The boundary layer separates beyond this critical value and also based solutions are not valid.

**Figure 3:**
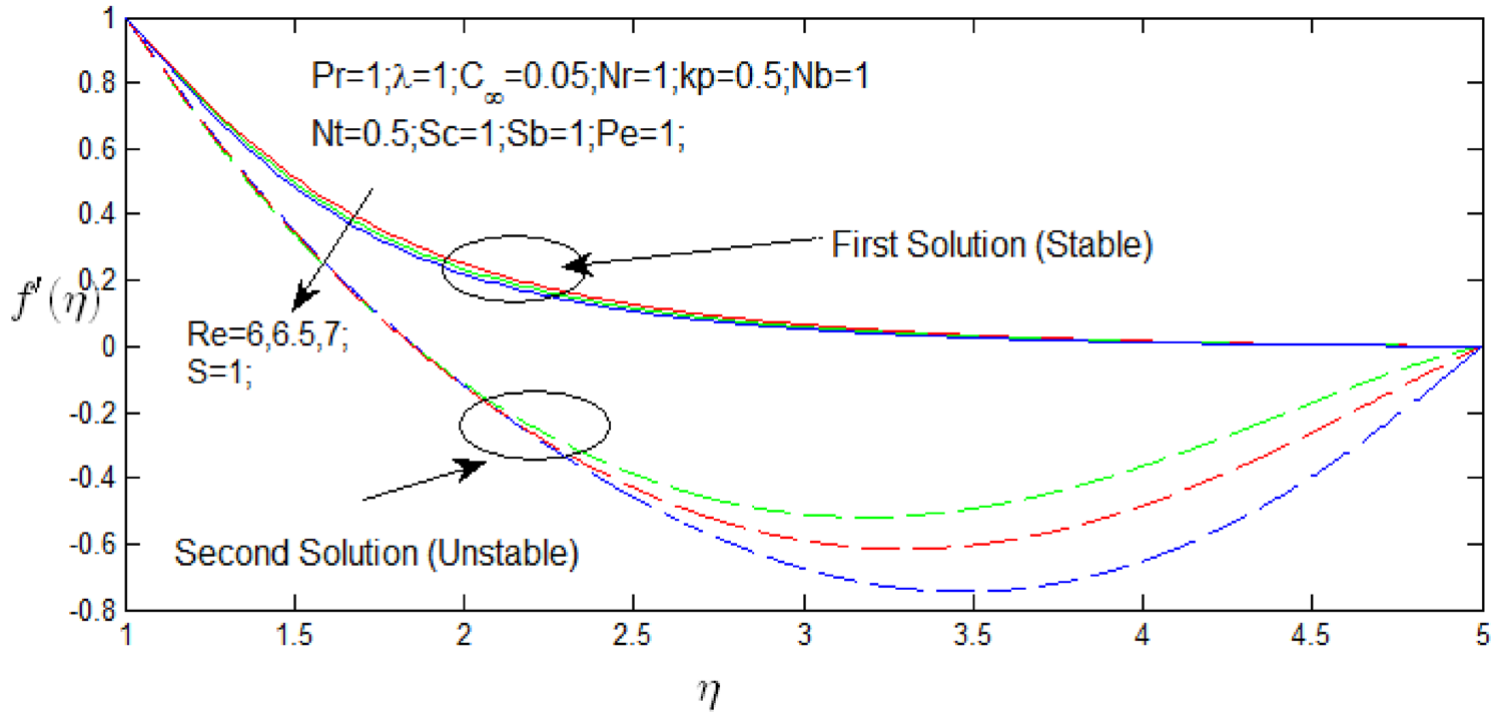
Velocity profile for different values of $Re$ when $s = 1$.

**Figure 4:**
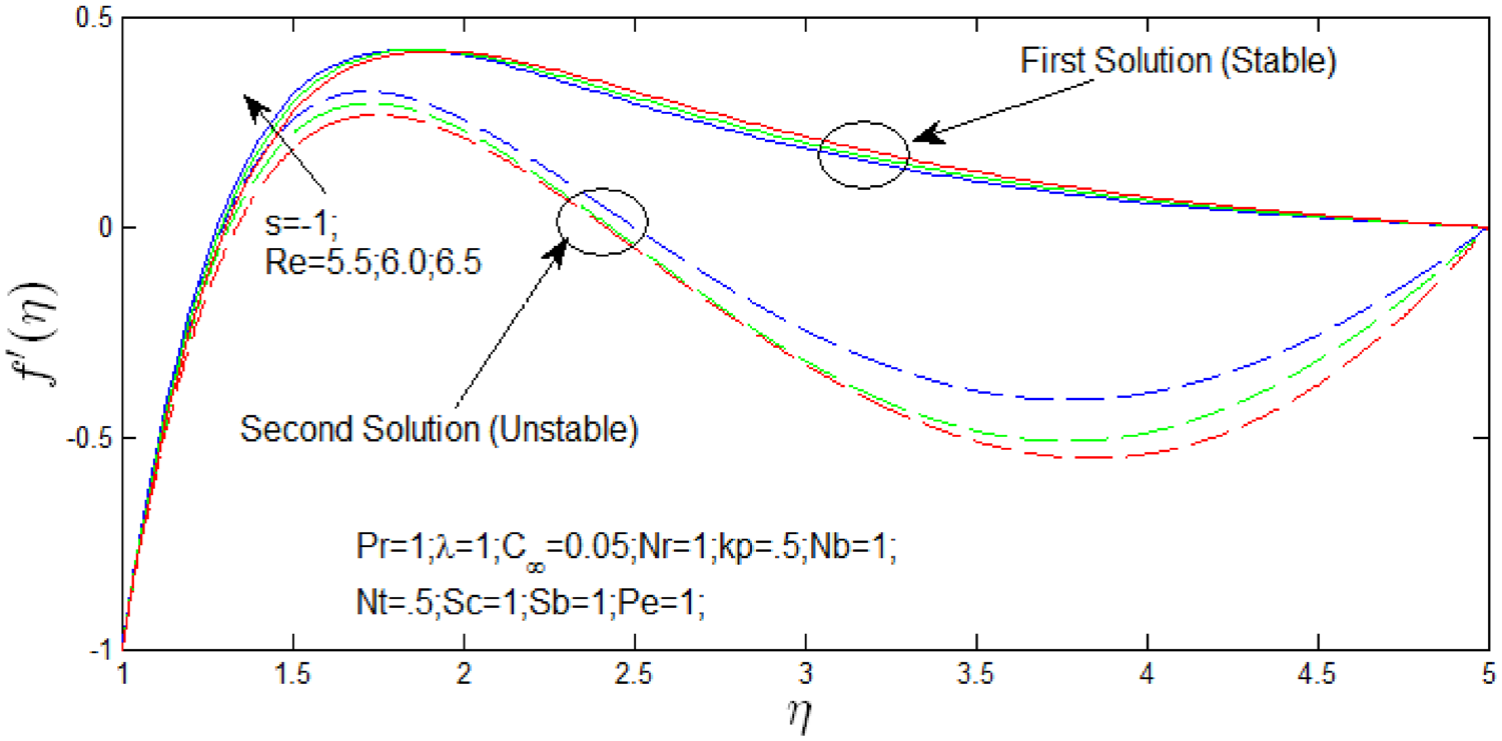
Velocity profile for different values of $Re$ when $s = - 1$.

In Figs. 3 and 4 illustrate the velocity profile $f^{\prime}\left( \eta \right)$ against $\eta$ for several values of $Re$ when $s = 1$ and $s = - 1$ respectively. The velocity profiles provide the existence of the dual solution when $s > s_{c}$ with diversity of $Re$. It also obtained that first solution is stable as the velocity profile went into positive range and the second solution is unstable as the velocity profile went out negative. Figures 3 and 4 show the influence of Reynolds number $Re$ over the dual solution. It is seen that increase in Reynolds number $Re$, the dual velocity profile decreases in Figs. 3 and 4 velocity profile decreases for first solution, but in second solution velocity profile increases. Although, the second solutions have negative values and unconditional there is no physical significance.

**Figure 5:**
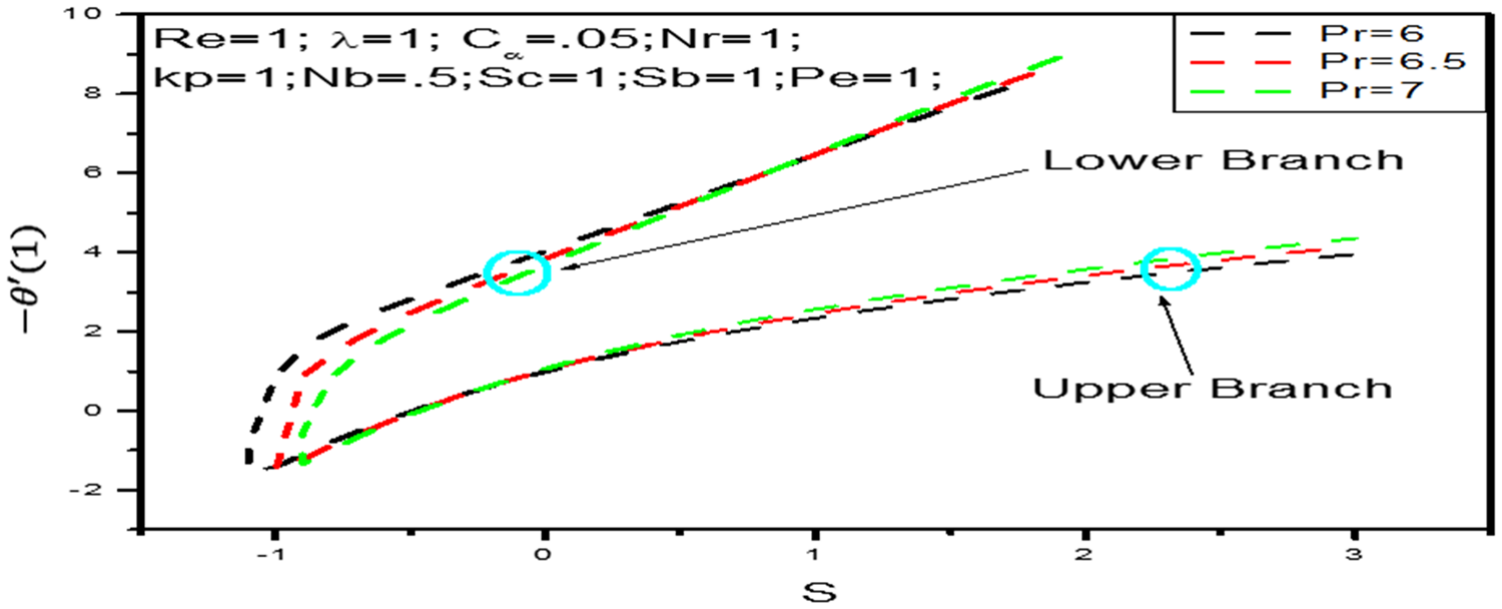
Local Nusselt number $- \theta ^{\prime}\left( 1 \right)$ with $s$ when $P_{r} = 6,6.5,7$

We variety the local Nusselt numbers are shown in Fig. 5 with respect to $s$ and variation of Prandtl number $P_{r}$. These Figure answer that it is possible to get dual solution of temperature profile when $s > - 1.1 = S_{c}$ and $P_{r} = 6$. Thus $s_{c}$ is the critical value for $P_{r} = 6$ and at this point only unique solution can be found. Also for $Pr = 6.5 \,\,{\mathrm{and}} P_{r} = 7$ the critical values are $s_{c} = - 1 \,\,{\mathrm{and}}\,\, s_{c} = - 0.9$ respectively. At this critical value both upper and lower branches are connected each other and at these point unique solutions exist. Behind from these critical values, boundary layer separates and the solution based on are not valid. It is found from heat transfer rate $- \theta ^{\prime}\left( 1 \right)$ increases strongly with parameter $s$ increases and relatively weakly with increasing Prandtl number $P_{r}$.

**Figure 6:**
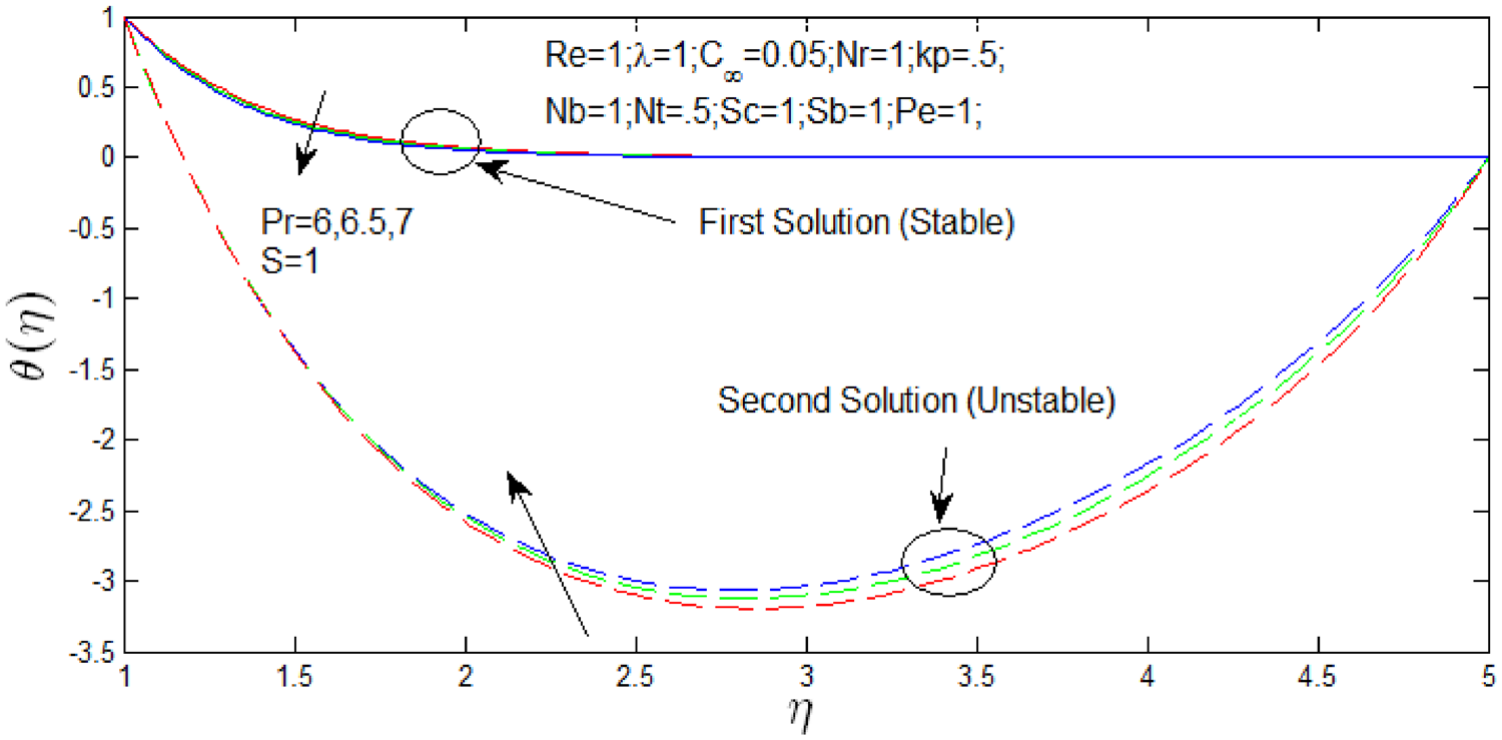
Heat profile for different values of $P_{r}$ and when $s = 1$.

**Figure 7:**
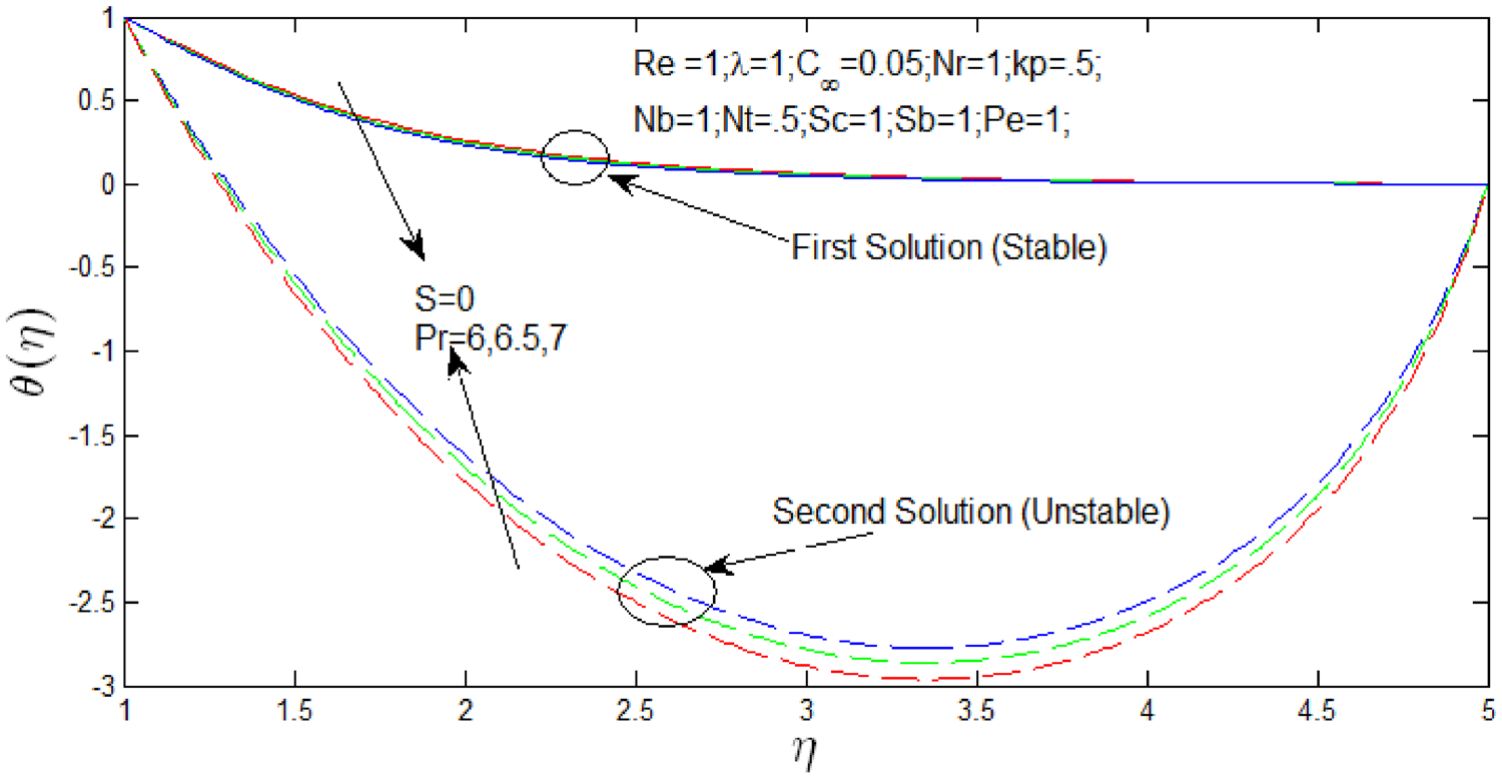
Heat profile for different values of $P_{r}$ and when $s = 0$.

The temperature profiles $\theta \left( \eta \right)$ against $\eta$ in Figs. 6 and 7 for different values of $P_{r}$ when $s = 1$ and $s = 0$ respectively. The temperature profiles that show the existence of the dual solution when $s > s_{c}$ with values of $Pr = 6,6.5,7$. It can be noticed that first solution remains stable as the temperature profile are in positive values and second solution is unstable as the temperature profile are in negative values. Figures 6 and 7 show the influence of $P_{r}$ over the dual velocity profile with Reynolds number $Re = 1$, $\lambda = 1$, $N_{r} = 1$, porosity parameter $k_{p} = 0.5$,$N_{b} = 1$, $N_{T} = .5$, schimidt number $S_{c} = 1$, bioconvection Schimidt number $S_{b} = 1$, and Peclet number $P_{e} = 1$. It is seen increase in Prandtl number $P_{r}$, the first solutions of temperature profiles decrease in Figs. 6 and 7, but temperature profiles increase for second solution. However, the second solutions of temperature have no importance if temperature profiles are in negative values and contradiction that $T_{\infty }$ is greater than boundary layer $T$.

**Figure 8:**
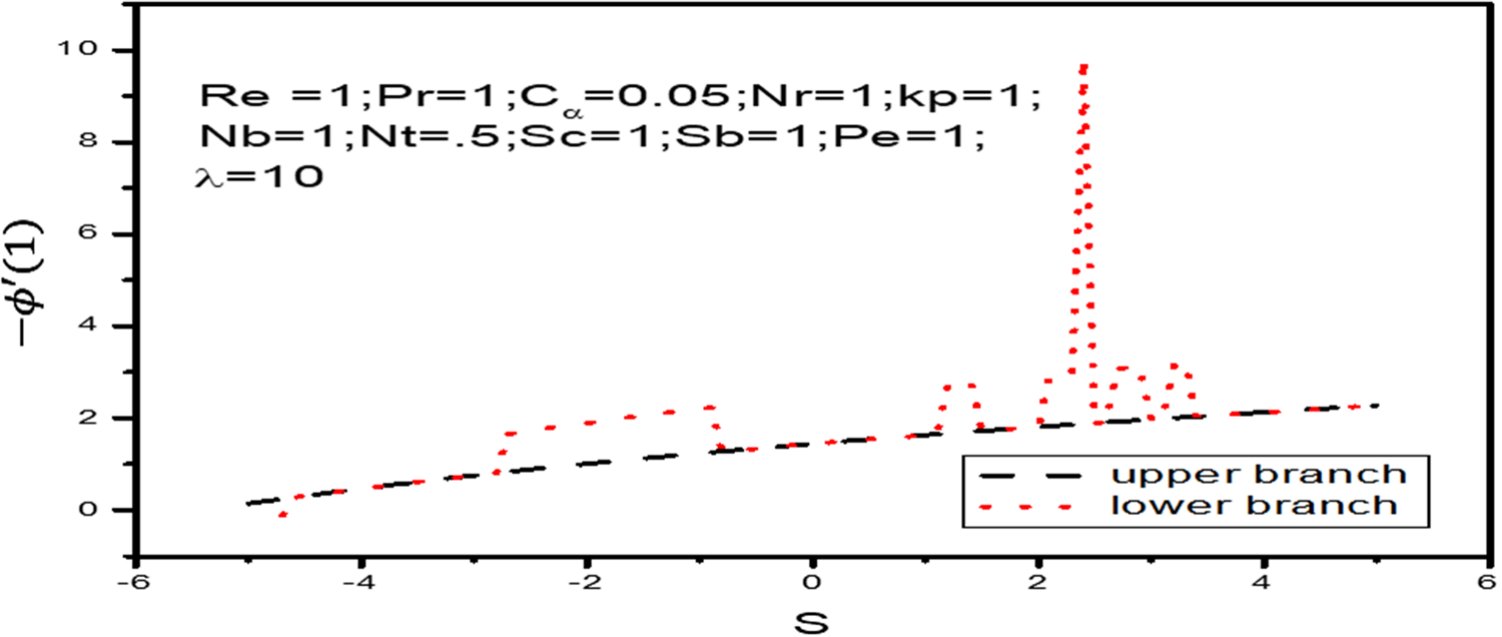
Variation of local nanoparticle mass transfer rate $- \phi ^{\prime}\left( 1 \right)$ as a function of $s$, when $\lambda = 10$.

Variation of local nanoparticle mass transfer rates are shown in Fig. 8 with respect to $s$ and natural convection parameter $\lambda$. These Figure shows that it is possible to get dual solution of nanoparticle concentration profile when $- 2.8 < s < - .7$ and also some points of $s > 0$ with $\lambda = 10$. Out of this critical range only unique solution can be found. At this critical value both upper and lower branches are connected each other and at these point unique solutions exist. Boundary layer separates behind the critical values and the solution based on it are not valid. It is found from that nanoparticle concentration rate $- \phi ^{\prime}\left( 1 \right)$ increases strongly as the parameter $s$ increases and relatively weakly with increasing natural convection parameter $\lambda$.

**Figure 9:**
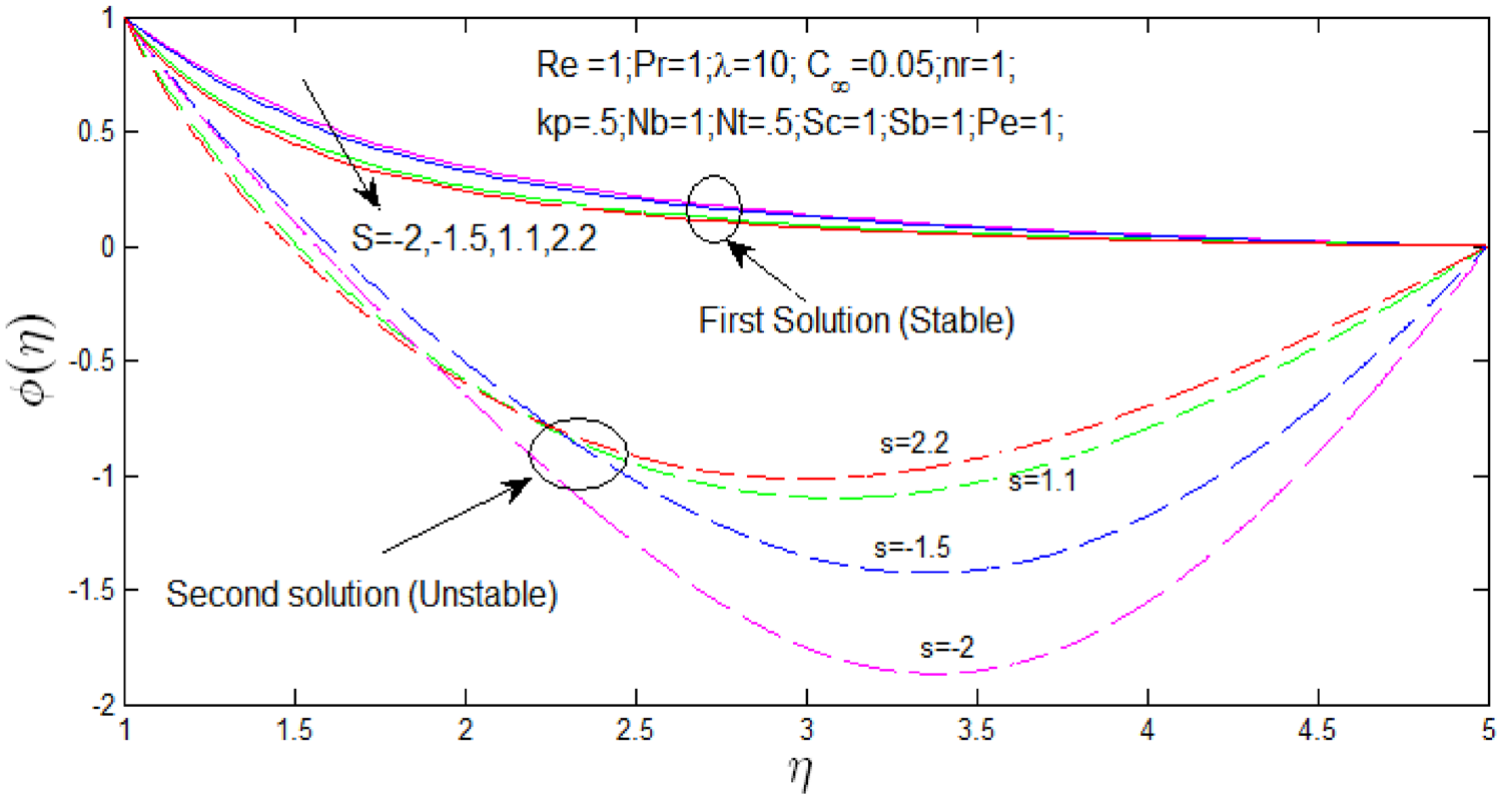
Nanoparticle concentration profile for different values of $s$ and when $\lambda = 10$.

**Figure 10:**
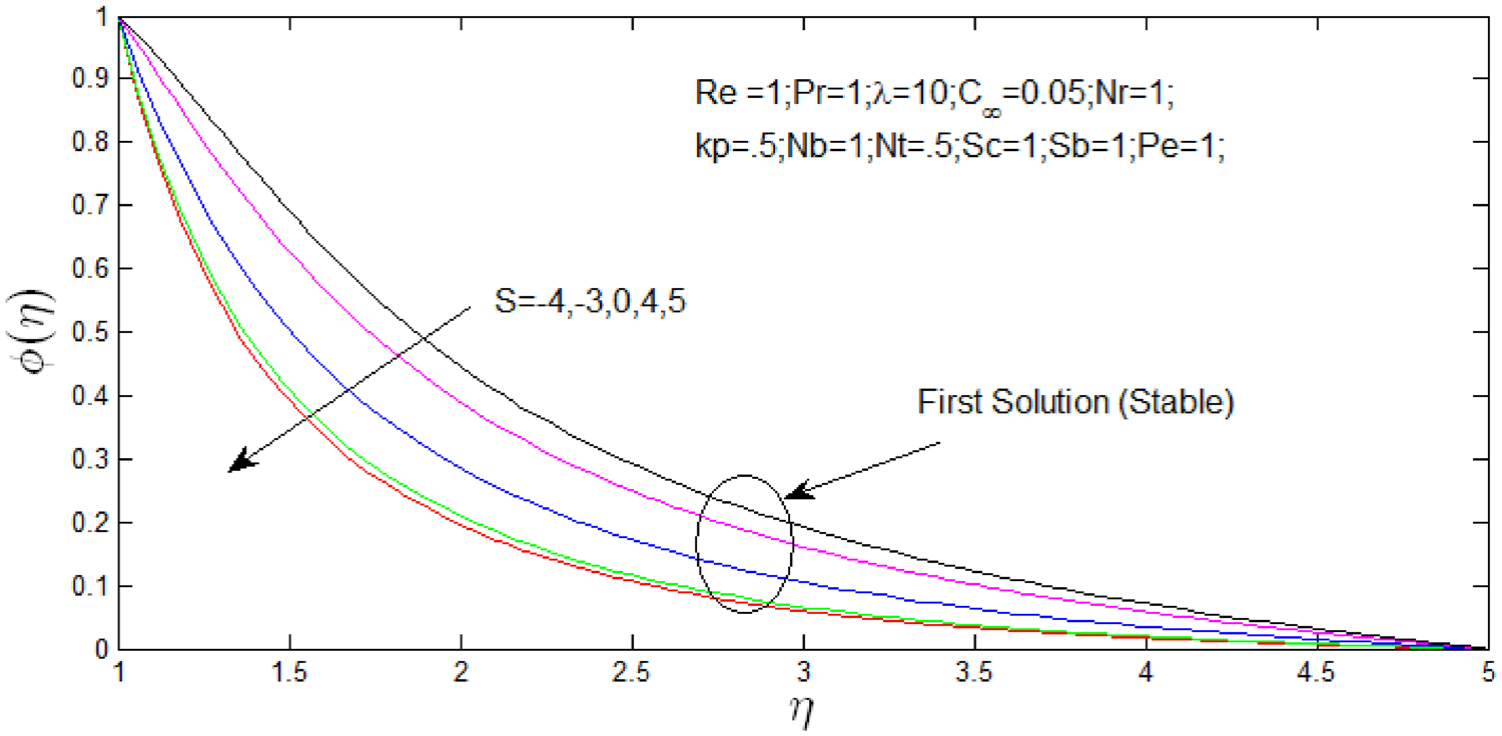
Nanoparticle concentration profile for different values of $s$ and with $\lambda = 10$.

Nanoparticle concentration profile $\phi \left( \eta \right)$ against $\eta$ in Fig. 9 for natural convection parameter $\lambda = 10$ with changing velocity ratio parameter $s$. Nanoparticle concentration profile that shown the existence of the dual solution when $s = - 2,\,\,s = - 1.5,\,\,s = 1.1 \,\,{\mathrm{and}} s = 2.2$ with values of $\lambda = 10$. It can be noticed that first solution is stable as the nanoparticle concentration profile are in positive range and the second solution is unstable as the nanoparticle concentration profile went out negative range. In Fig. 10 nanoparticle concentration profile that shown that dual solutions did not exist when $s = - 4,\,\,s = - 3,\,\,s = 0,\,\,s = 4 \,\,{\mathrm{and}} s = 5$ with values of $\lambda = 10$ and unique stable solution can be found. Figures 9 and 10 show the influence of $s$ over the dual and unique nanoparticle concentration profile when the nanoparticle concentration ratio $0.05\%$, Reynolds number $Re = 1$, Prandtl number $P_{r} = 1,\lambda = 10$, buoyancy ratio $N_{r} = 1$, porosity parameter $k_{p} = 0.5$, $N_{b} = 1$, $N_{T} = 0.5$, schimidt number $S_{c} = 1$, bioconvection Schimidt number $S_{b} = 1$, and Peclet number $P_{e} = 1$. It is seen that increase in velocity ratio parameter $s$ the first solutions of nanoparticle concentration profiles decrease in Figs. 9 and 10, but nanoparticle concentration profiles increase for second solution in Fig. 9.

**Figure 11:**
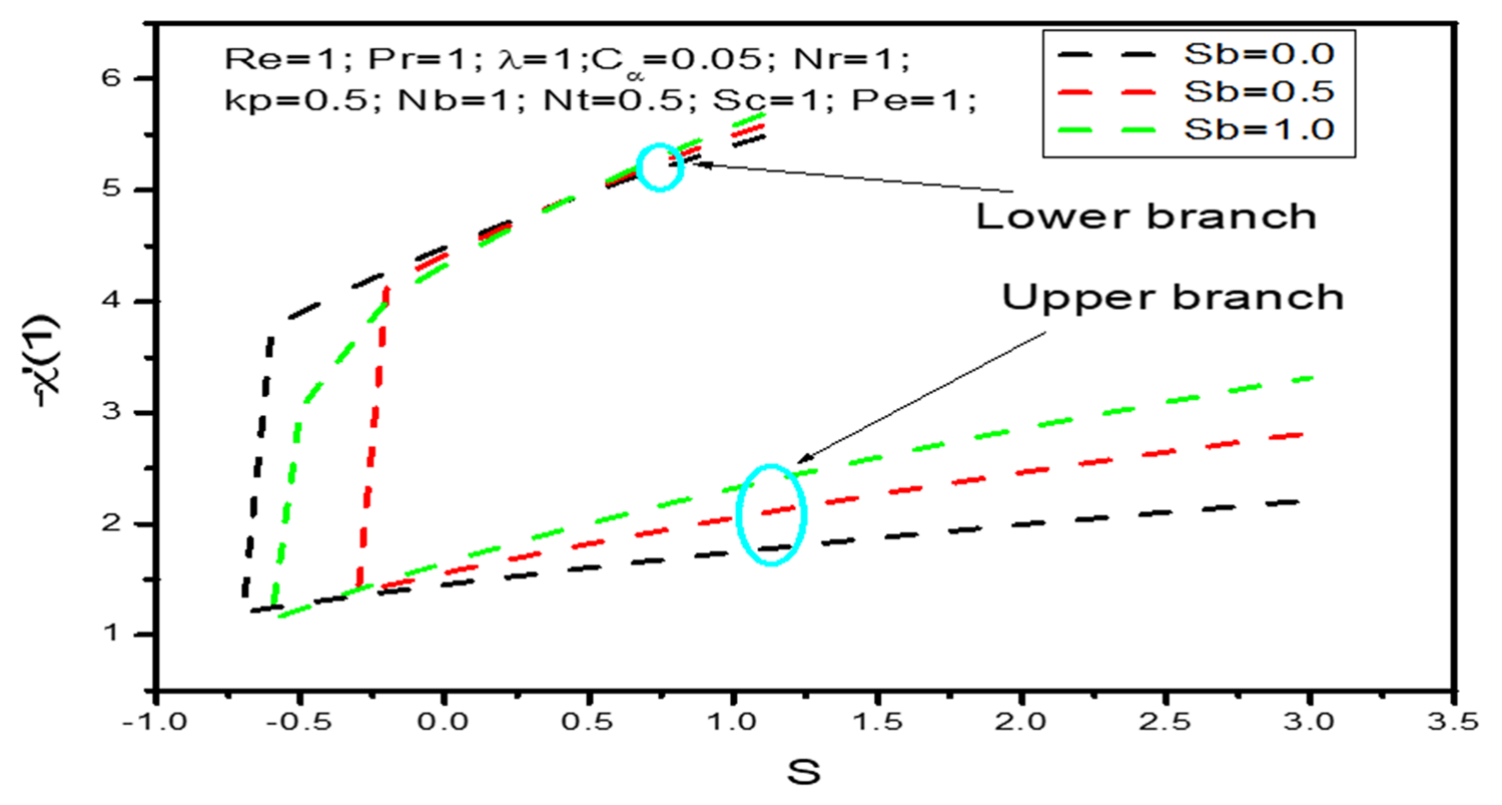
The density number of microorganism $- \chi ^{\prime}\left( 1 \right)$ as a function of $s$ when $Sb = 0,0.5,1$

Variation of density number of microorganism are shown in Fig. 11 with respect to $s$ and with variation of bioconvection Schimidt $S_{b}$. These figure shows that it is possible to get dual solution of density number of microorganism profile when $s > - 0.7 = s_{c}$ with $S_{b} = 0$. Thus $s_{c}$ is the critical value for $S_{b} = 0$ and at this point only unique solution can be found. Also for $S_{b} = 0.5\,\,{\text{ and}}\,\,\,S_{b} = 1$ the critical values are $s_{c} = - 0.6\,\,{\text{ and}}\,\,\, s_{c} = - 0.3$ respectively. At this critical values both upper and lower branches are connected each other and at these point and unique solutions exist and behind at this critical value unique solution exist. Boundary layer separates behind the critical values and the solution based upon are not valid. It is found from that density number of microorganism $- \chi ^{\prime}\left( 1 \right)$ increases strongly as the parameter $s$ increases and relatively weakly with increasing bioconvection Schimidt number $S_{b}$.

**Figure 12:**
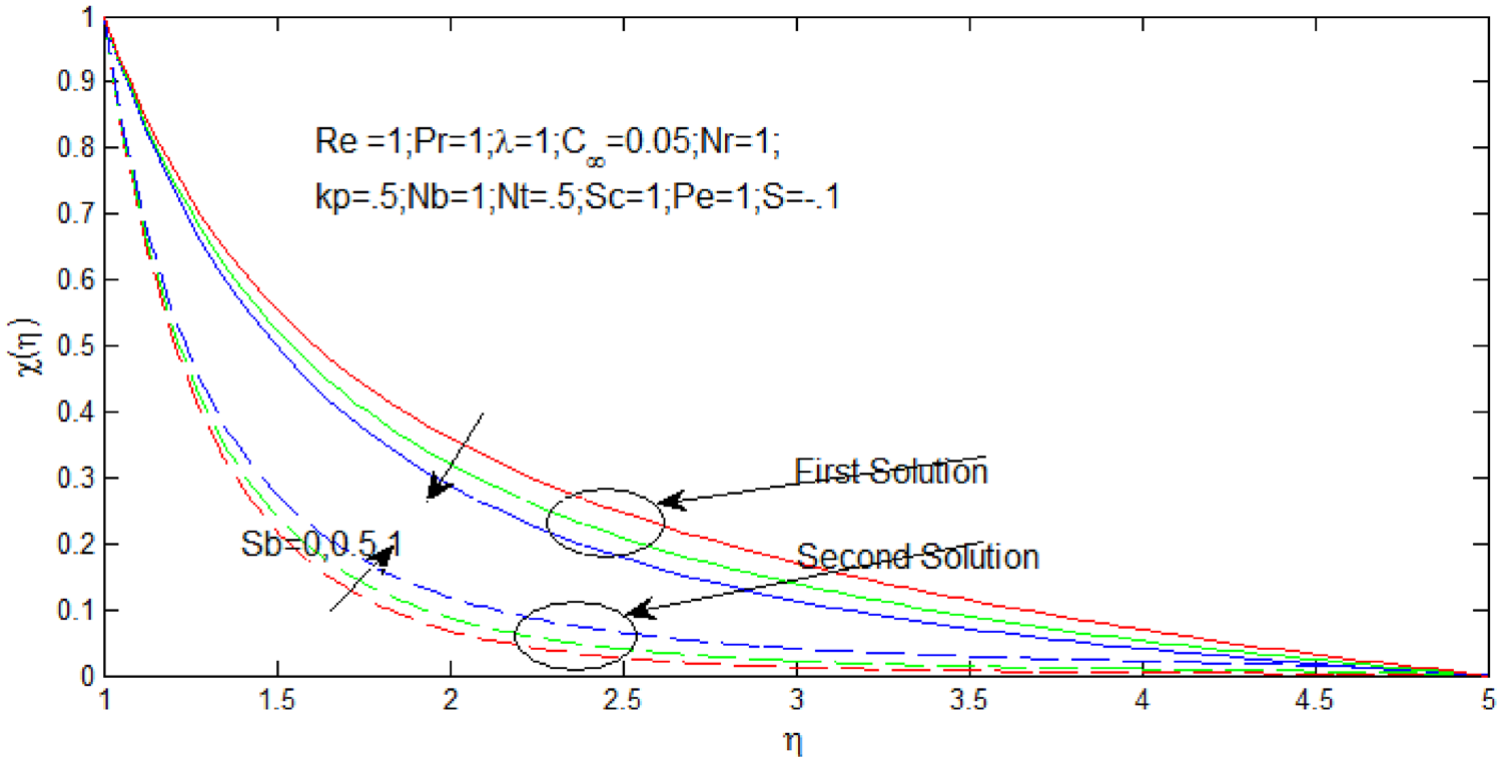
Density of microorganism profile for different values of $S_{b}$ with $s = - .1$

**Figure 13:**
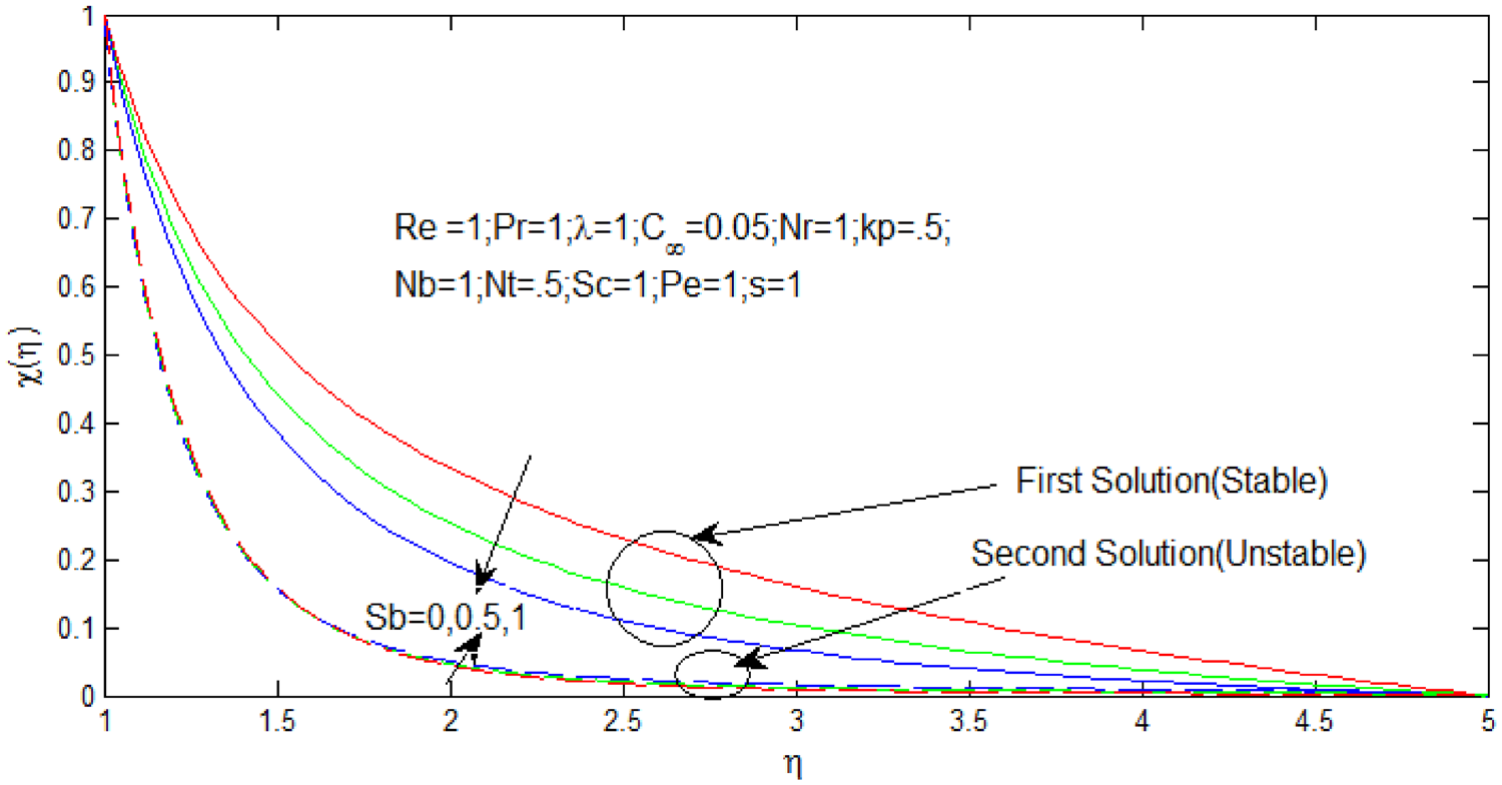
Density of microorganism profile for different values of $S_{b}$ when $s = 1$.

The microorganism density profiles $\chi \left( \eta \right)$ against $\eta$ in Figs. 12 and 13 for several values of $S_{b}$ when $s = - 0.1$ and $s = 1$ respectively. The temperature profiles that shown the existence of the dual solution when $s > s_{c}$ with values of $S_{b} = 0,\,\,0.5,\,\,1$. It can be noticed that first solution of microorganism density is stable as the velocity and temperature profiles are in positive range and the second solution of microorganism density profile is unstable as the velocity and temperature profile went out negative range. Figures 17 and 18 show the influence of bioconvection Schimidt number $S_{b}$ over the dual velocity profile when the nanoparticle concentration ratio $0.05\%$, Reynolds number $Re = 1$, Prandtl number $Pr = 1$, $\lambda = 1$, $N_{r} = 1$, porosity parameter $k_{p} = .5$, $N_{b} = 1$, $N_{T} = .5$, Schimidt number $S_{c} = 1$, and Peclet number $P_{e} = 1$. It is obtained that increase in bioconvection Schimidt number $S_{b}$, the first solutions of microorganism density profiles decrease in Figs. 12 and 13, but microorganism density profiles increase for second solution.

**Figure 14:**
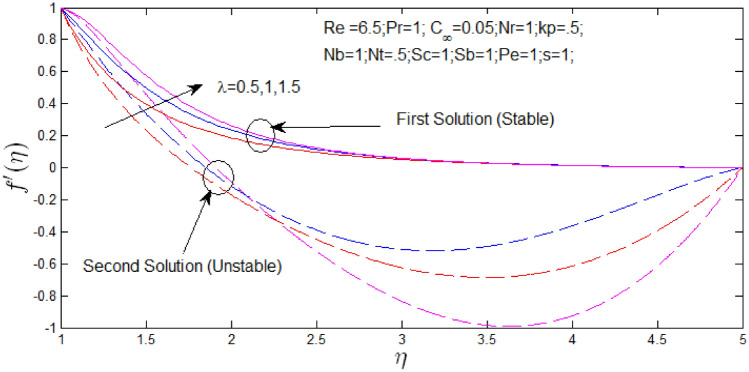
Velocity profile for different values of $\lambda$ with $s = 1$.

**Figure 15:**
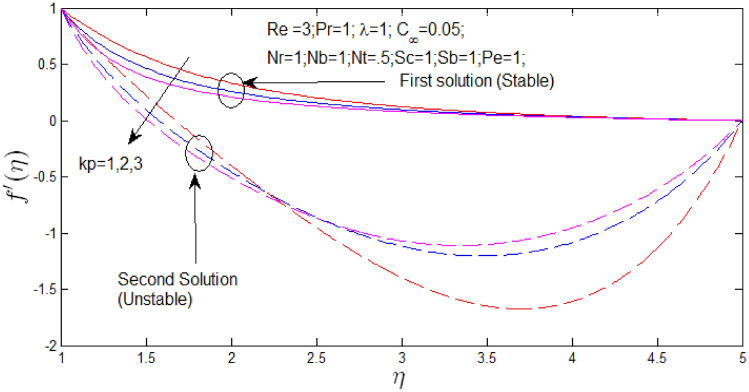
Velocity profile for different values of $k_{p}$ with $s = 1$.

The velocity profile $f^{\prime}\left( \eta \right)$ against $\eta$ for several values of $\lambda$ and $k_{p}$ in Figs. 14 and 15 respectively when $s = 1$. The velocity profiles provide the existence of the dual solution with $s = 1$ with certain change of natural convection parameter $\lambda$ and porosity parameter $k_{p}$. It can be obtained first solution is stable as the velocity profile went into positive range and the second solution is unstable as the velocity profile are in negative values. Figure 14 shown the effect of $\lambda$ over the dual solution when the nanoparticle concentration ratio is $0.05\%$, Reynolds number $Re = 6.5$, Prandtl number $Pr = 1$, porosity parameter $k_{p} = 0.5$, $N_{r} = 1$, $N_{b} = 1$, $N_{T} = 0.5$, Schmidt number $S_{c} = 1$, bioconvection Schmidt number $S_{b} = 1$, and Peclet number $Pe = 1$. It is observed increase in parameter $\lambda$, the dual velocity profile increase. Figure 15 shown the effect of $k_{p}$ over the dual solution with Reynolds number $Re = 3$, Prandtl number $Pr = 1$, natural convection parameter $\lambda = 1N_{r} = 1$, $N_{b} = 1$, $N_{T} = 0.5$, Schmidt number $S_{c} = 1$, bioconvection Schmidt number $S_{b} = 1$, and Peclet number $Pe = 1$. It is concluded that increase in $k_{p}$, the dual velocity profile decrease.

**Figure 16:**
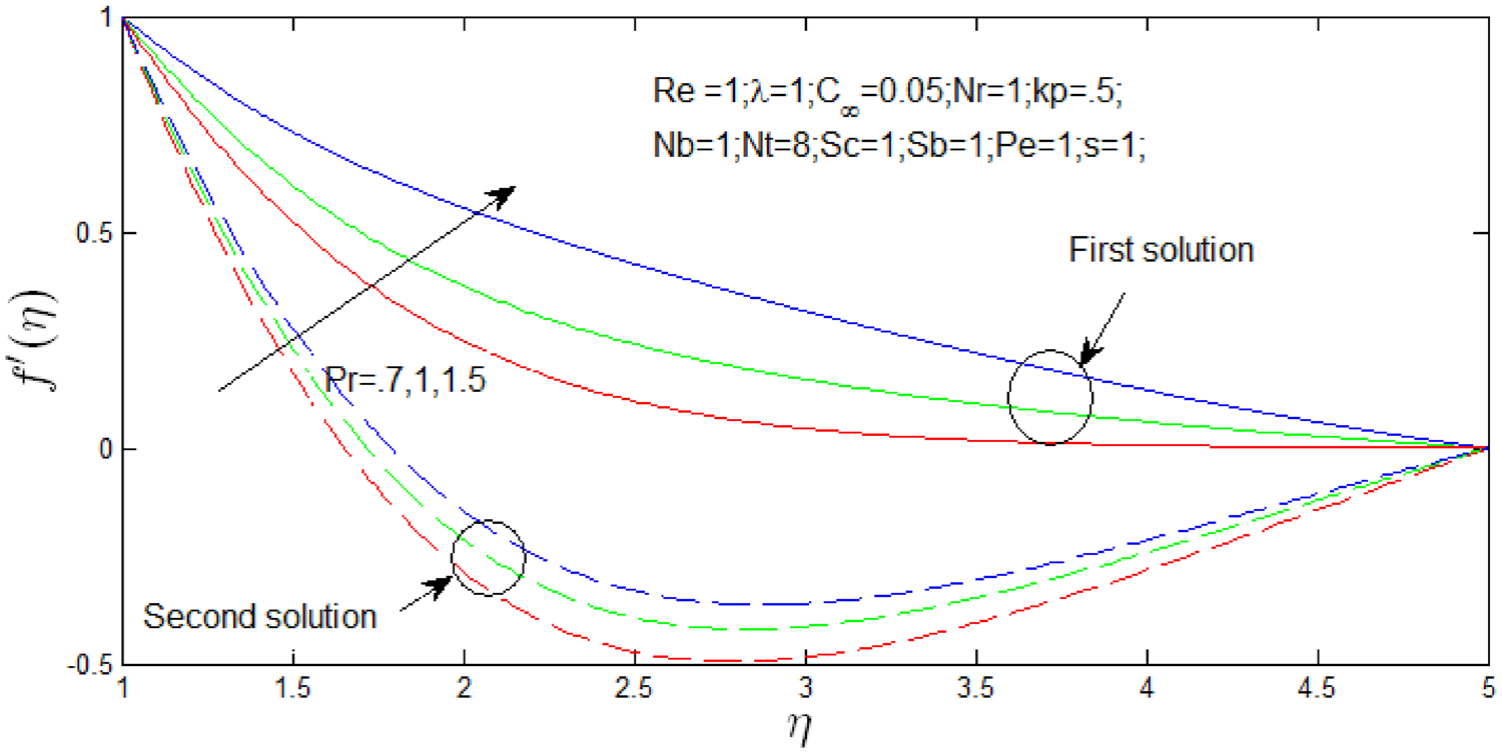
Velocity profile for different values of $Pr$ with $s = 1$.

**Figure 17:**
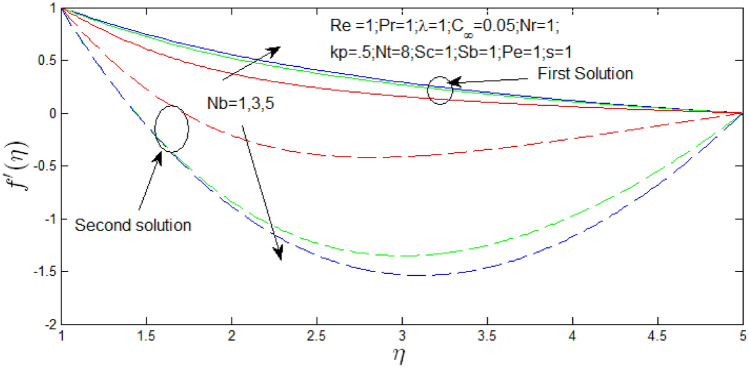
Velocity profile for different values of $Nb$ with $s = 1$.

The velocity profile $f^{\prime}\left( \eta \right)$ against $\eta$. for several values of $Pr$ and $N_{b}$ in Figs. 16 and 17 respectively when $s = 1$. The velocity profiles declared the existence of the dual solution when $s = 1$ with certain change of $Pr$ and Brownian motion parameter $N_{b}$. It can be seen first solution is stable as the velocity profiles went into positive values and the second solution is unstable as the velocity profiles are negative values. Figure 16 shown the influence of Prandtl number $Pr$ over the dual velocity profiles with Reynolds number $Re = 1$, natural convection parameter $\lambda = 1$, porosity parameter $k_{p} = 0.5$, $N_{r} = 1$, $N_{b} = 1$, $N_{T} = 8$, Schmidt number $S_{c} = 1$, bioconvection Schmidt number $S_{b} = 1$, d Peclet number $Pe = 1$. It is seen increase in $Pr$, the dual velocity profiles increase. Figure 17 shown the influence of Brownian motion parameter $N_{b}$ over the dual velocity profiles when Reynolds number $Re = 1$, Prandtl number $Pr = 1$, natural convection parameter $\lambda = 1$ buoyancy ratio $N_{r} = 1$, porosity parameter $k_{p} = .5$, thermophoresis parameter $N_{T} = 8$, Schmidt number $S_{c} = 1$, bioconvection Schmidt number $S_{b} = 1$, and Peclet number $Pe = 1$. It is concluded that increase in $N_{b}$, the velocity profiles of first solutions increase and second solution decrease.

**Figure 18:**
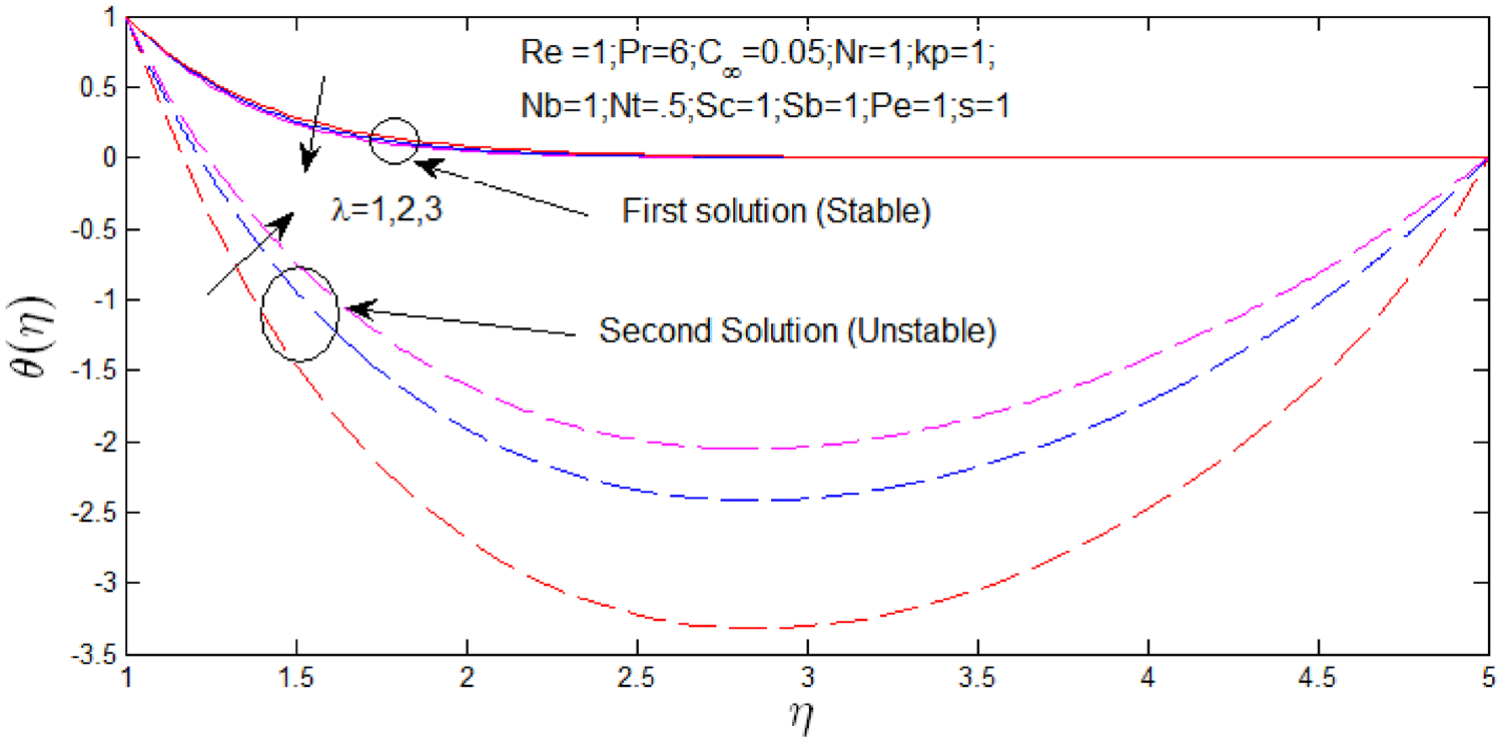
Temperature profile for different values of $\lambda$ with $s = 1$.

The temperature profile $\theta \left( \eta \right)$ against $\eta$ for several values of $\lambda$ in Fig. 18 with $s = 1$. The temperature profiles that show the existence of the dual solution when h^$s = 1$ with certain change of natural convection parameter $\lambda$. It can be seen that first solution is stable as the temperature profiles went into positive values and the second solution is unstable as the temperature profiles are in negative values. Figure 18 shown the influence of $\lambda$ over the dual solution with Reynolds number $Re = 1$, $Pr = 6$, porosity parameter $k_{p} = 0.5$, $N_{r} = 1$, $N_{b} = 1$, $N_{T} = .5$, Schmidt number $S_{c} = 1$, bioconvection Schmidt number $S_{b} = 1$, and Peclet number $Pe = 1$. It is seen increase in $\lambda$, the first solution decreases and second solution increases.

**Figure 19:**
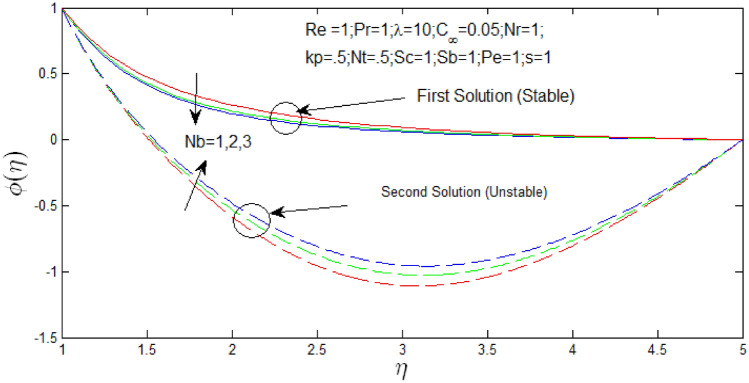
Nanoparticle concentration profile for different values of $N_{b}$ with $s = 1$.

Figure 19 shown the influence of $N_{b}$ over the dual solutions of nanoparticle concentration with Reynolds number $Re = 1$, Prandtl number $Pr = 1$, porosity parameter $k_{p} = 0.5$, buoyancy ratio $N_{r} = 1$, natural convection parameter $\lambda = 10$, thermophoresis parameter $N_{T} = .5$, Schmidt number $S_{c} = 1$, bioconvection Schmidt number $S_{b} = 1$, and Peclet number $Pe = 1$. It is seen increase in $N_{b}$, the first solution decreases, but second solution increases.

**Figure 20:**
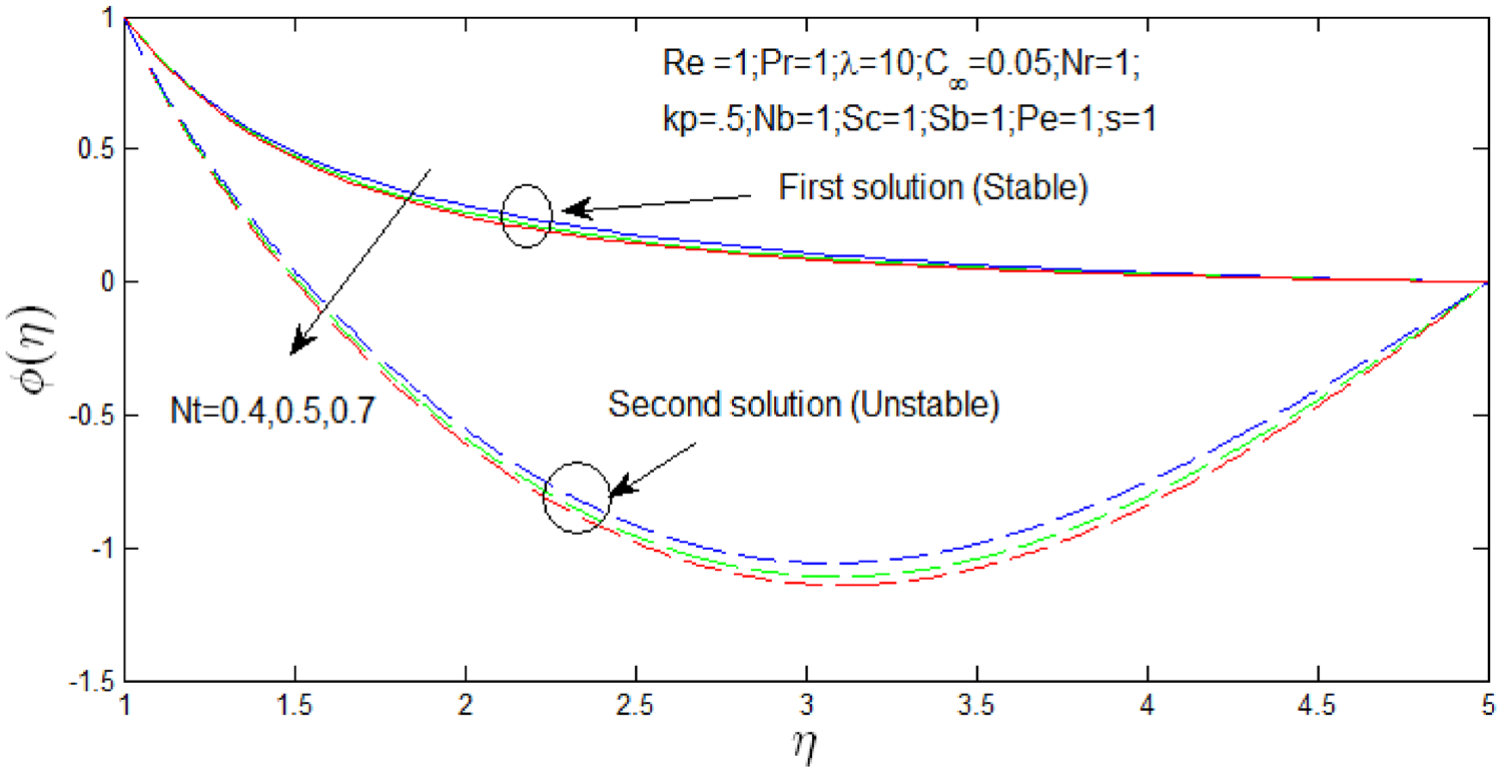
Nanoparticle concentration profile for different values of $N_{T}$ with $s = 1$.

Figure 20 shown the influence of $N_{t}$ over the dual solutions of nanoparticle concentration with Reynolds number $Re = 1$, Prandtl number $Pr = 1$, natural convection parameter $\lambda = 1$, porosity parameter $k_{p} = 0.5$, buoyancy ratio $N_{r} = 1$, Brownian motion parameter $N_{b} = 1$, Schmidt number $S_{c} = 1$, bioconvection Schmidt number $S_{b} = 1$, and Peclet number $Pe = 1$. It is seen increase in $N_{T}$, the first solution decreases and second solution decreases.

**Figure 21:**
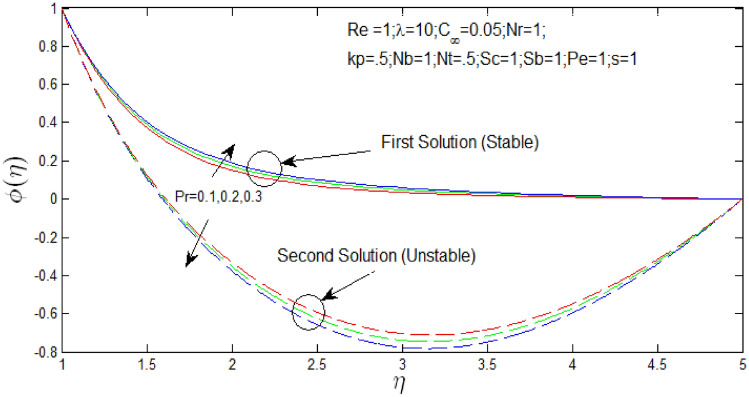
Nanoparticle concentration profile for different values of $Pr$ with $s = 1$.

Figure 21 shown the effect of $Pr$ over the dual solutions of nanoparticle concentration with Reynolds number $Re = 1$, natural convection parameter $\lambda = 10$ porosity parameter $k_{p} = 0.5$, buoyancy ratio $N_{r} = 1$, natural convection parameter $\lambda = 10$, thermophoresis parameter $N_{T} = .5$, Brownian motion parameter $N_{b} = 1$, Schmidt number $S_{c} = 1$, bioconvection Schmidt number $S_{b} = 1$, and Peclet number $Pe = 1$. It is seen increase in $N_{b}$, the first solution decreases, but second solution increases.

**Figure 22:**
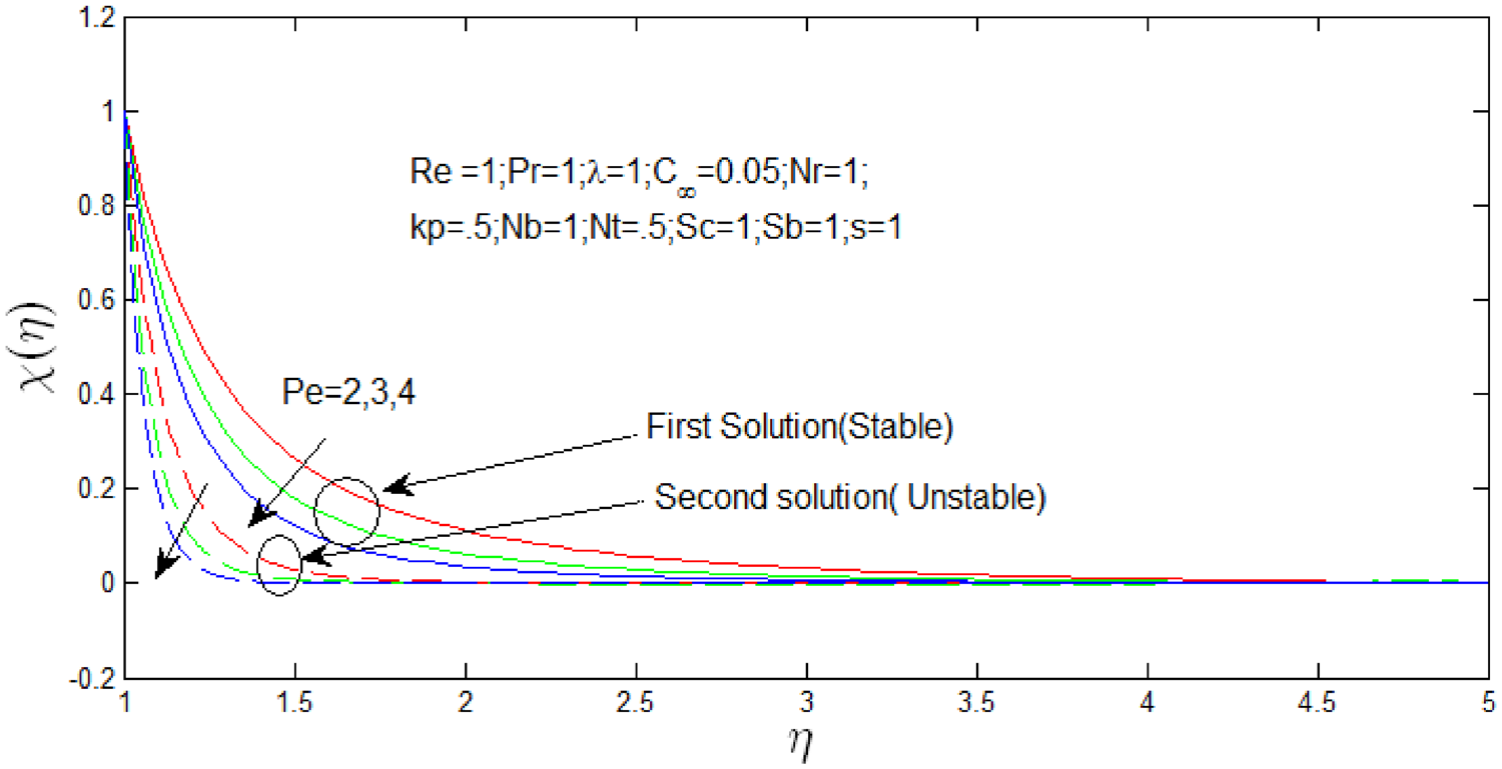
Density of microorganism profile for different values of $Pe$ with $s = 1$.

Figure 22 shown the influence of Peclet number $Pe$ over the dual solutions of density of microorganism when Reynolds number $Re = 1$, Prandtl number $Pr = 1$, porosity parameter $k_{p} = 0.5$, buoyancy ratio $N_{r} = 1$, natural convection parameter $\lambda = 10$, Brownian motion parameter $N_{b} = 1$ thermophoresis parameter $N_{T} = .5$, Schmidt number $S_{c} = 1$, bioconvection Schmidt number $S_{b} = 1$. It is observed that increase in Peclet number $Pe$, the first solution decreases, and second solution decreases.

**Figure 23:**
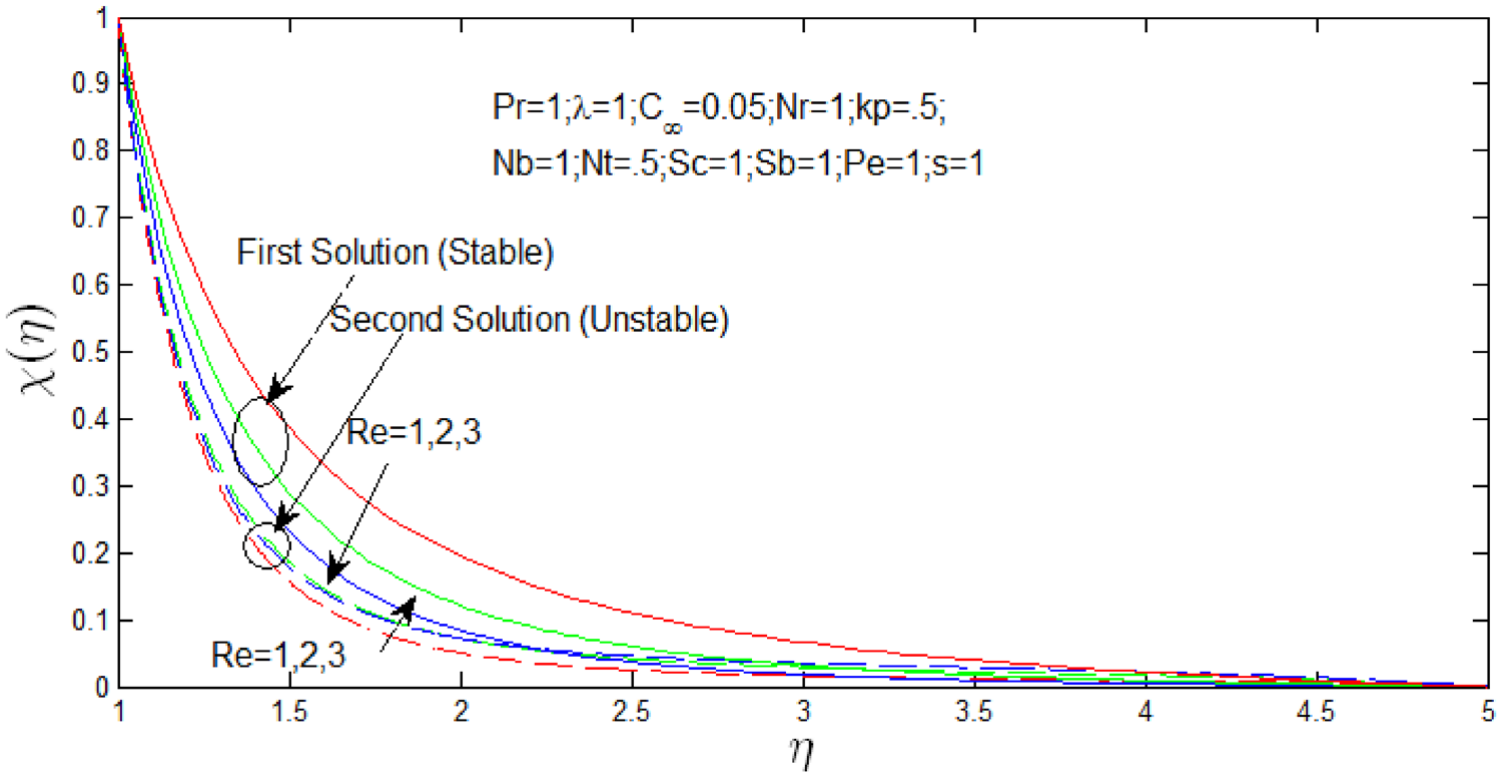
Density of microorganism profile for different values of $Re$ with $s = 1$.

Figure 23 shown the influence of Reynolds number $Re$ over the dual solutions of density of microorganism when Prandtl number $Pr = 1$, natural convection parameter $\lambda = 1$, porosity parameter $k_{p} = 0.5$, $N_{r} = 1$, $N_{b} = 1$, $N_{t} = 0.5$, Schmidt number $S_{c} = 1$, bioconvection Schmidt number $S_{b} = 1$, and Peclet number $Pe = 1$. It is observed that increase in Reynolds number $Re$, the first solution decreases and second solution increases.

**Figure 24:**
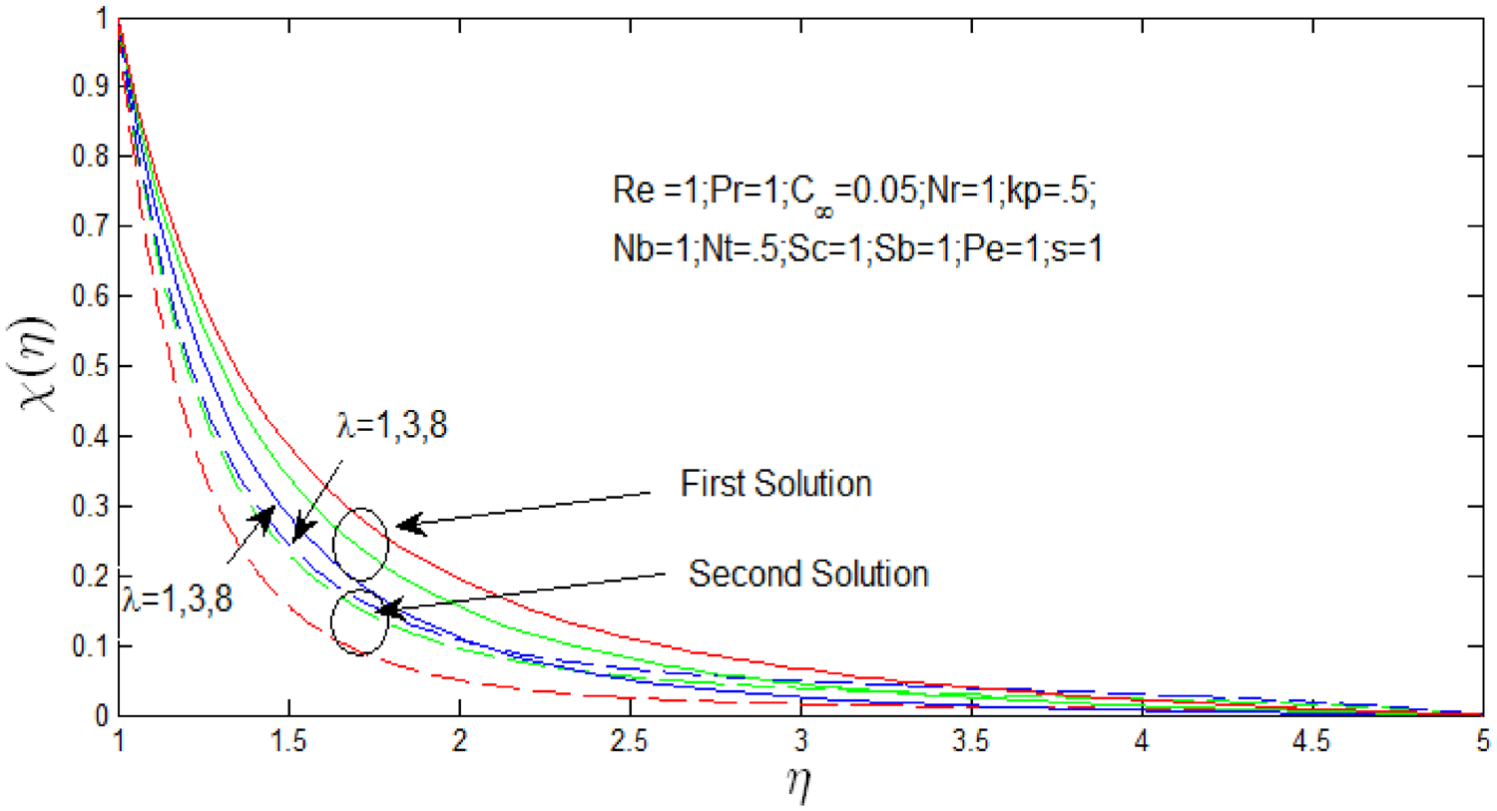
Density of microorganism profile for various values of $\lambda$ when $s = 1$.

Figure 24 shown the effect of $\lambda$ over the dual solutions of density of microorganism whenReynolds number $Re = 1$, Prandtl number $Pr = 1$, porosity parameter $k_{p} = 0.5$, buoyancy ratio $N_{r} = 1$, thermophoresis parameter $N_{T} = .5$, Brownian motion parameter $N_{b} = 1$, Schmidt number $S_{c} = 1$, bioconvection Schmidt number $S_{b} = 1$, and Peclet number $Pe = 1$. It is seen increase in $\lambda$, the first solution decreases, but second solution increases.

### Validation of results by comparison

Validation of the solutions via comparison related to previous simpler models is included in Table 1 and Table 2. To verify the fluency of the present study, we compared the result with reported by Rehman et al.^35^ and Faisal et al.^36^, and show good rapport.

**Table 1:** Comparison of local skin friction coefficient $- f^{\prime\prime}\left( 1 \right)$ at the cylindrical surface for several values of Reynolds number when $Pr = 5,N_{r} = 1,\lambda = 0.5, N_{t} = 0.5,N_{b} = 1,S_{c} = 1 \,\,\,{\text{and }}\,\,C_{\infty } = 0.01$.

Reynolds number,$Re$	Rehman et al.^37^	Present study (first solution)
0.5	0.4579	0.442586
1	0.8392	0.807715
3	1.6903	1.660243
5	2.2383	2.213786
7	2.6729	2.652396

**Table 2:** Comparison of local Nusselt number $- \theta ^{\prime}\left( 1 \right)$ at the cylindrical surface for several values of Prandtl number when $Re = 3,\,\,N_{r} = 1,\,\,\lambda = 1, \,\,N_{t} = 0.5, \,\,N_{b} = 0.5,S_{c} = 1 \,\,{\text{and }}\,\,C_{\infty } = 0.01$.

Prandtl number,$Pr$	Rehman et al.^37^	Present study (first solution)
0.71	1.1647	1.223106
1	1.4482	1.504346
3	2.8423	2.923257
5	3.8627	3.947500
7	4.7102	4.794861

## Conclusions

In this works, we are numerically investigated the problem of boundary layer flow, heat transfer, nanoparticle concentration and density of microorganism over vertical stretching cylinder addressing the effect of velocity ratio, natural convection and porosity medium. The transforming boundary layer equations were solved by build in function bvp4c in MATLAB. Results indicate that multiple solutions exist. Critical point separated upper branch and lower branch solutions. Stable solutions were indicated by upper branch and unstable solutions were also indicated by lower branch. Effects of velocity ratio parameter, Reynolds number Prandtl number, natural convection parameter, Schmidt number, buoyancy ratio, Peclet number, local skin friction coefficient, local Nusselt number, local nanoparticle mass transfer rate and local density number of microorganisms have been examined. The key finding briefing as bellow:

- From the variation of skin friction coefficient the dual velocity profiles existed some critical values when $s > - 2.9 = s_{c} ,s > - 2.5 = s_{c} \,\,\,{\mathrm{and}}\,\, s > - 2.2 = s_{c}$ respectively for $Re = 7,\,\,6.5,\,\,6$.
- The variation of local Nusselt number shows that it is possible to get the dual solutions of temperature profile when the critical value $s > - 1.1 = s_{c} , \,\,s_{c} = - 1 \,\,{\mathrm{and}}\,\,\,s_{c} = - 0.9$ respectively for $P_{r} = 6,\,\,6.5,\,\,7$.
- The variation of local nanoparticle mass transfer rate shows the dual solutions of nanoparticle concentration profile arise when $- 2.8 < s < - 0.7$ and some points of $s > 0$ with $\lambda = 10$.
- Variation of density number of microorganism provides the dual solutions of microorganism density profile initiate when $s > - 0.7 = s_{c} ,\,\,\, s_{c} = - 0.6\,\,{\text{ and}} s_{c} = - 0.3$ respectively for $S_{b} = 0,\,\,0.5 \,\,{\text{and }}\,\,\,1$.
